# Right ventricular energetic biomarkers from 4D Flow CMR are associated with exertional capacity in pulmonary arterial hypertension

**DOI:** 10.1186/s12968-022-00896-8

**Published:** 2022-12-01

**Authors:** Xiaodan Zhao, Shuang Leng, Ru-San Tan, Ping Chai, Tee Joo Yeo, Jennifer Ann Bryant, Lynette L. S. Teo, Marielle V. Fortier, Wen Ruan, Ting Ting Low, Ching Ching Ong, Shuo Zhang, Rob J. van der Geest, John C. Allen, Marina Hughes, Pankaj Garg, Teng Hong Tan, James W. Yip, Ju Le Tan, Liang Zhong

**Affiliations:** 1 National Heart Centre Singapore, National Heart Research Institute Singapore, Singapore, Singapore; 2 Duke-NUS Medical School, Singapore, Singapore; 3 National University Hospital Singapore, Singapore, Singapore; 4 Yong Loo Lin School of Medicine, National University of Singapore, Singapore, Singapore; 5 KK Women’s and Children’s Hospital, Singapore, Singapore; 6 Singapore Institute for Clinical Sciences, A*STAR, Singapore, Singapore; 7 Philips Healthcare Germany, Hamburg, Germany; 8 Department of Radiology, Leiden University Medical Center, Leiden, Netherlands; 9 Department of Cardiovascular Medicine, University of East Anglia, Norwich, UK

**Keywords:** 4D flow CMR, Flow components, Kinetic energy, Cardiopulmonary exercise test, Pulmonary arterial hypertension

## Abstract

**Background:**

Cardiovascular magnetic resonance (CMR) offers comprehensive right ventricular (RV) evaluation in pulmonary arterial hypertension (PAH). Emerging four-dimensional (4D) flow CMR allows visualization and quantification of intracardiac flow components and calculation of phasic blood kinetic energy (KE) parameters but it is unknown whether these parameters are associated with cardiopulmonary exercise test (CPET)-assessed exercise capacity, which is a surrogate measure of survival in PAH. We compared 4D flow CMR parameters in PAH with healthy controls, and investigated the association of these parameters with RV remodelling, RV functional and CPET outcomes.

**Methods:**

PAH patients and healthy controls from two centers were prospectively enrolled to undergo on-site cine and 4D flow CMR, and CPET within one week. RV remodelling index was calculated as the ratio of RV to left ventricular (LV) end-diastolic volumes (EDV). Phasic (peak systolic, average systolic, and peak E-wave) LV and RV blood flow KE indexed to EDV (KEI_EDV_) and ventricular LV and RV flow components (direct flow, retained inflow, delayed ejection flow, and residual volume) were calculated. Oxygen uptake (VO_2_), carbon dioxide production (VCO_2_) and minute ventilation (VE) were measured and recorded.

**Results:**

45 PAH patients (46 ± 11 years; 7 M) and 51 healthy subjects (46 ± 14 years; 17 M) with no significant differences in age and gender were analyzed. Compared with healthy controls, PAH had significantly lower median RV direct flow, RV delayed ejection flow, RV peak E-wave KEI_EDV_, peak VO_2_, and percentage (%) predicted peak VO_2_, while significantly higher median RV residual volume and VE/VCO_2_ slope. RV direct flow and RV residual volume were significantly associated with RV remodelling, function, peak VO_2_, % predicted peak VO_2_ and VE/VCO_2_ slope (all *P* < 0.01). Multiple linear regression analyses showed RV direct flow to be an independent marker of RV function, remodelling and exercise capacity.

**Conclusion:**

In this 4D flow CMR and CPET study, RV direct flow provided incremental value over RVEF for discriminating adverse RV remodelling, impaired exercise capacity, and PAH with intermediate and high risk based on risk score. These data suggest that CMR with 4D flow CMR can provide comprehensive assessment of PAH severity, and may be used to monitor disease progression and therapeutic response.

Trial registration number: https://www.clinicaltrials.gov. Unique identifier: NCT03217240.

**Supplementary Information:**

The online version contains supplementary material available at 10.1186/s12968-022-00896-8.

## Introduction

Pulmonary arterial hypertension (PAH) is a progressive disease characterized by elevated pulmonary vascular resistance (PVR) and increase in pulmonary arterial pressure, which leads to progressive right ventricular (RV) failure, a major cause of morbidity and mortality [1]. Cardiovascular magnetic resonance (CMR) is the reference standard for cardiac volume, function, mass and blood flow quantification due to its high accuracy and reproducibility, and has become obligatory for follow-up of patients with PAH [2, 3].

4D flow CMR, an emerging time-resolved phase-contrast technique with flow velocities encoded simultaneously in three directions allows comprehensive measurement of blood flow dynamics in the heart and large vessels with full volumetric coverage throughout the cardiac cycle [4–6]. It has been applied to investigate pulmonary artery hemodynamics in patients with PAH [7–12] but studies on intracardiac blood flow are scarce. Employing 4D flow CMR, Han et al. [13] demonstrated altered RV kinetic energy (KE) parameters derived from computational analysis of individual pathlines and velocities within the RV between PAH patients and controls. More recently, Wang et al. [14] demonstrated reduction in RV direct flow and increase in RV residual volume proportions in PAH patients compared with controls, in addition to negative and positive associations of RV direct flow and RV residual volume, respectively, with PVR.

Exercise intolerance is a cardinal symptom of PAH that impacts quality of life and survival [15]. The cardiopulmonary exercise test (CPET) quantifies the cardiovascular, respiratory, metabolic and muscular response to physical effort and provides an objective assessment of functional capacity and limitation [16]. In PAH, CPET enables prognostic risk stratification and informs clinical decisions on the need and timing of therapeutic interventions [17].

Heretofore, no study has investigated the association of 4D flow CMR with exercise capacity in PAH. This study aimed to compare 4D flow components and KE-derived parameters between PAH patients and healthy controls, and investigate their associations with RV remodelling, RV functional and CPET outcomes.

## Methods

### Study population

**Fig. 1:**
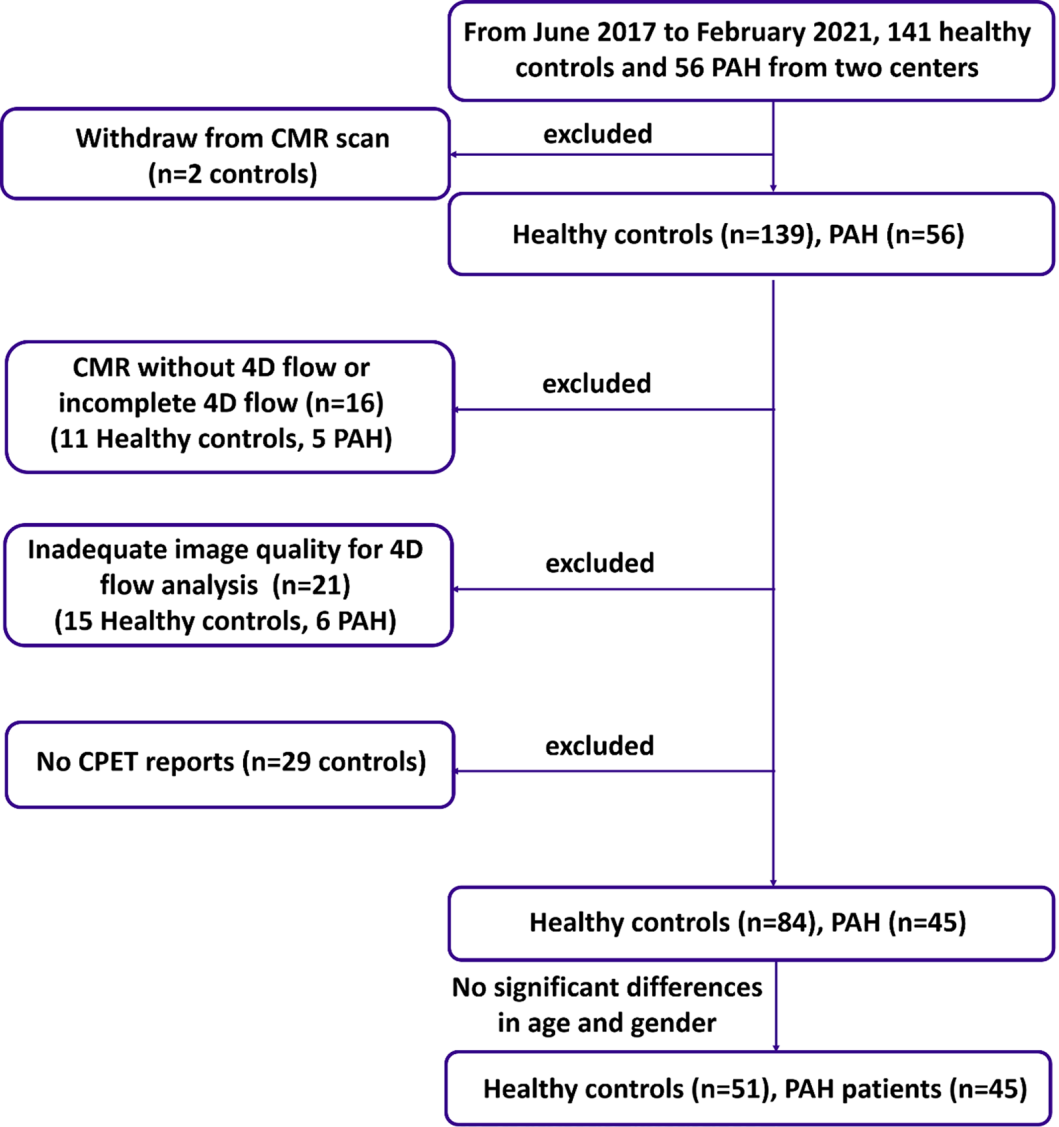
Flowchart of subject recruitment. *4D* four-dimensional, *PAH* pulmonary arterial hypertension, *CMR* cardiovascular magnetic resonance, *CPET* cardiopulmonary exercise test

PAH patients and healthy subjects in this prospective study were identified from the image database of INITIATE study, which was a multicenter registry of healthy volunteers and participants with congenital heart disease (*ClinicalTrials.gov* identifier NCT03217240). The study protocol had been approved by site institutional review boards. Written informed consent was obtained from all subjects. Part of the study population was included in previous studies to investigate the impact of age, sex and ethnicity on left ventricular (LV) flow components and kinetic energy [18], and associations of 4D flow parameters with RV functional, remodelling and CPET outcome in repaired tetralogy of Fallot patients [19]. From June 2017 to February 2021, 56 PAH patients and 141 healthy subjects were recruited. After applying the exclusion criteria (Fig. 1), 45 PAH patients (F/M: 38:7) and 51 healthy controls (F/M: 34:17) with no significant differences in age and gender were included in the final analysis. Our PAH group comprised 23 patients with idiopathic PAH; 4 with heritable PAH; 13 with connective tissue disease (CTD), which included systemic sclerosis, mixed CTD, systemic lupus erythematosus, Sjogren syndrome and anti-synthetase syndrome; and 5 with congenital heart disease (CHD), which included repaired and unrepaired atrial septal defect, and pulmonary atresia with ventricular septal defect.

In addition, according to the risk stratification strategy proposed by Kylhammar et al. [20], clinical variables including World Health Organization (WHO) functional class, six-minute walk test (6MWT), N-terminal pro-brain natriuretic peptide, maximal right atrial (RA) area from CMR, mean right arterial pressure (RAP) and cardiac index from right heart catheterization (RHC), peak oxygen uptake (VO_2_) and slope of the ratio of minute ventilation (VE) to carbon dioxide production (VCO_2_) from CPET, were individually graded 1 to 3 where 1 = “low risk”, 2 = “intermediate risk”, and 3 = “high risk”. Overall risk was quantified as the mean grade, i.e., the sum of all grades divided by the number of grades rounded to the nearest integer. Moreover, REVEAL 2.0 score was calculated based on the risk score calculator [21]. Clinical outcomes were analysed in all patients with PAH including death and PAH-related hospitalization.

### Acquisition of Cardiovascular magnetic resonance images

CMR was performed using standardized protocols on 3 T CMR system (Ingenia, Philips Healthcare, Best, the Netherlands) and 1.5 T CMR system (Magneton Aera, Siemens Healthineers, Erlangen, Germany) scanners, respectively, as previously published [18, 19]. Standard two-, three- and four-chamber long-axis and stacks of short-axis views covering both LV and RV were acquired with end-expiratory breath hold balanced steady-state free precession sequences. Whole-heart 4D flow CMR was performed during free breathing without respiratory navigator in accordance with recommendations [5, 6]. Typical 4D flow acquisition parameters were previously reported in [18, 19] and we provided these in Additional file 1: Table S1.

### Cardiovascular magnetic resonance image analysis

All analyses were performed at a core laboratory.

#### Cine image analysis

LV mass, LV and RV volumes were analyzed offline using MASS software (version 2019EXP, Leiden University Medical Center, Leiden, The Netherlands) using the protocol previously published [18, 19]. RV mass was calculated from end-diastolic short-axis stack of ventricular images [22]. RV remodelling index was defined as the ratio of RV end-diastolic volume (RVEDV) to LV end-diastolic volume (LVEDV) [23] and a value exceeding mean + 3SD (calculated from healthy controls) connoted adverse RV remodelling. RA area was planimetered on the four-chamber long axis cine CMR in the time frame just before the opening of the tricuspid valve. In the four-chamber long-axis plane, we semi-automatically tracked the deformation of the medial and lateral tricuspid valve insertions as well as the lateral mitral valve insertion with reference to the epicardium of the cardiac apex using published semi-automatic fast feature-tracking methodology [24–27]. The inter-ventricular synchrony index was defined as the time difference to maximal displacement between the tracked deformations at the lateral mitral and tricuspid valve insertions. Maximal displacement―tricuspid annular plane systolic excursion (TAPSE) in ventricular systole―was determined from the displacement curve generated by the lateral tricuspid valve insertion point on the four-chamber long-axis plane. Fast RV strain assessment was performed as previously published [27], and RV fast global longitudinal strain (GLS) —strain value at end-systole—was obtained from the strain curve. Main pulmonary artery (PA) relative area change was quantified as$100{\%}\text{x}(\text{m}\text{a}\text{x}\text{i}\text{m}\text{a}\text{l} \text{P}\text{A} \text{a}\text{r}\text{e}\text{a} - \text{m}\text{i}\text{n}\text{i}\text{m}\text{a}\text{l} \text{P}\text{A} \text{a}\text{r}\text{e}\text{a})/\text{m}\text{i}\text{n}\text{i}\text{m}\text{a}\text{l} \text{P}\text{A} \text{a}\text{r}\text{e}\text{a}$, where PA areas were obtained by semi-automatically tracking the PA cross-section contours on the magnitude images of the 2D phase-contrast readouts in MASS software [28, 29].

#### 4D flow analysis

4D flow CMR images were analyzed using MASS software. The analysis techniques and definitions of both LV and RV four flow components (direct flow, retained inflow, delayed ejection flow, residual volume) and KE normalized to EDV parameters (peak systole, average systole and peak E-wave KEI_EDV_) were the same with our previous publication [18, 19], and the details were provided in Additional file 2. KE discordance was defined as the ratio of RV to LV average systolic KEI_EDV_, and fractional flow ratio was defined as the ratio of RV direct flow to RV residual volume. Movies with appropriate color legend for four RV flow components in one healthy control subject, and one PAH were provided in Additional file 3.

### Cardiopulmonary exercise test

All subjects underwent CPET at a central laboratory within one week after CMR. The protocol was provided in our previous publication [19]. VE, VO_2_ and VCO_2_ were acquired breath-by-breath and averaged over ten seconds. Peak VO_2_ was the highest 10-second averaged sample obtained during exercise; percentage (%) predicted peak VO_2_ was calculated by indexing peak VO_2_ against normative values [30, 31]; VE/VCO_2_ slope was calculated via least squares linear regression (y = mx + b, m = slope) using VE and VCO_2_ values acquired from the start of exercise to peak. Based on a cut-off value of 15 ml/kg/min, patients were stratified into two groups: preserved peak VO_2_ (> 15 ml/kg/min); and abnormal peak VO_2_ (≤ 15 ml/kg/min) [16]. Separately, % predicted peak VO_2_ ≤ 65% and/or VE/VCO_2_ slope ≥ 36 was selected for patients at intermediate and high risks in terms of exercise capacity [2, 32]. For the seven PAH patients who could not complete full CPET, default CPET values (peak VO_2_ = 14 ml/kg/min; % predicted peak VO_2_ = 46% and VE/VCO_2_ slope = 45) were recorded [33].

### Reproducibility

20 subjects were randomly selected for reproducibility evaluation. To assess intraobserver variability, a second segmentation of ventricular contours was performed by the primary observer (XDZ) one month after initial segmentation. To assess interobserver variability, LV and RV endocardial and epicardial contours were segmented by a second independent observer (SL) blinded to the first observer’s results.

### Statistical analysis

Data were analyzed using SPSS (version 23.0, Statistical Package for the Social Sciences, International Business Machines, Inc., Armonk, New York, USA). All continuous variables were presented as mean ± standard deviation (SD) or median (interquartile range = 75th percentile−25th percentile) where applicable. Associations between continuous variables were investigated using linear regression and Pearson correlation analyses. Comparison of means between two groups was analyzed using two-sample *t* test for normally distributed data, Mann-Whitney U tests for non-normally distributed data; and Kruskal-Wallis (K-W) non-parametric one-way ANOVA for more than two groups with post-hoc pair-wise comparisons in the event of a significant K-W test with Bonferroni corrections where appropriate. Bonferroni significance levels were calculated for the comparisons of RV 4D flow and CPET parameters between controls and PAH. For secondary and exploratory results, only raw P-values were reported and it is left to the reader to apply any desired significance level calculation. Univariate and multivariate regression analyses were performed to investigate the determinants of adverse RV remodelling (RVEDV/LVEDV ratio), right ventricular ejection fraction (RVEF) and CPET outcomes (% predicted peak VO_2_). Variables that showed significant associations (*P* < 0.05) on univariate analyses were input as independent variables for multivariate linear regression analyses. Regression residuals were assessed visually for variance homogeneity and via Q-Q plots for normality. Receiver operator characteristic (ROC) analyses were performed to assess the discriminative value of RV measurements and area under ROC curve (AUC) used as a measure of discriminative capability. Youden’s indexes were defined for all points of the ROC curve and the maximum value used as the criterion for selecting the optimum threshold point [34]. *Post hoc* Delong test was used to assess the statistical differences between AUCs. A nested binary logistic regression analysis was used to investigate incremental value of RV direct flow. 4D flow CMR parameters as risk factors for the composite adverse clinical outcome were investigated using Cox proportional-hazards regression analysis with hazard ratios adjusted for age, gender, and body surface area. Intra- and inter-observer reproducibility results were assessed using the intra-class correlation coefficient (ICC), the paired-sample *t* test and coefficients of variation, and agreement using Bland-Altman analysis. Statistical significance was set at *P* < 0.05.

## Results

### Subject characteristics and ventricular function

Baseline characteristics for patients and controls are tabulated in Table 1. Mean ages for PAH patients and healthy controls were 46 ± 11 and 46 ± 14 years, respectively. PAH patients were shorter and had higher mean body mass index and heart rate than healthy controls (all *P* < 0.05). PAH patients had significantly higher indexed RVEDV and RV end-systolic volume (RVESV), increased RV mass and RV/LV mass ratio, and reduced RVEF and PA relative area change compared with healthy controls but there were no significant differences in indexed LV mass, LVEDV, LV end-systolic volume (LVESV) and LV stroke volume (SV) between two groups. The remodelling index (RVEDV/LVEDV) was 0.97 ± 0.10 among healthy controls; accordingly, a value exceeding 1.27 (mean + 3SD) connoted adverse RV remodelling. Compared with healthy controls, PAH patients exhibited significantly reduced TAPSE and RV GLS, and dilated RA (all *P* ≤ 0.001, Table 1). Mean interventricular synchrony index was significantly longer in PAH patients than healthy controls (0 (0, 32) ms versus 30 (0, 48) ms, *P* = 0.001) (Additional file 4: Fig. S1). Compared with PAH patients with low risk, PAH patients with intermediate and high risk based on both risk score and REVEAL 2.0 score had greater RVESV index, and lower RVEF and RV GLS (Additional file 1: Table S2).

**Table 1:** Baseline characteristics of healthy controls and pulmonary arterial hypertension (PAH)

	Healthy control (n = 51)	PAH (n = 45)	*P*
Demographics and clinical parameters			
Age, years	46 ± 14	46 ± 11	0.828
Sex, M/F	17/34	7/38	0.059
Height, cm	163 ± 8	159 ± 8	**0.017**
Weight, kg	60 ± 11	61 ± 13	0.515
Body surface area, m^2^	1.64 ± 0.17	1.64 ± 0.19	0.935
Body mass index, kg/m^2^	22.4 ± 3.1	24.1 ± 4.6	**0.032**
Systolic blood pressure, mmHg	126 ± 19	119 ± 20	0.074
Diastolic blood pressure, mmHg	75 ± 13	71 ± 14	0.087
Heart rate, bpm	71 ± 14	82 ± 16	**0.001**
NT–proBNP, pg/mL*	**–**	207 (337)	**–**
Functional class > WHO I, n (%)	**–**	18 (40%)	**–**
Types of PAH			
Idiopathic PAH, n (%)	**–**	23 (51%)	**–**
Heritable PAH, n (%)	**–**	4 (9%)	**–**
PAH associated with connective tissue disease, n (%)	**–**	13 (29%)	**–**
Systemic sclerosis, n (%)	**–**	4 (31%)	**–**
Mixed connective tissue disease, n (%)	**–**	3 (23%)	**–**
Systemic lupus erythematosus, n (%)	**–**	3 (23%)	**–**
Sjogren syndrome, n (%)	**–**	2 (15%)	**–**
Anti–synthetase syndrome, n (%)	**–**	1 (8%)	**–**
PAH associated with congenital heart disease, n (%)	**–**	5 (11%)	**–**
Atrial septal defect repaired, n (%)	**–**	3 (60%)	**–**
Atrial septal defect unrepaired, n (%)	**–**	1 (20%)	**–**
Pulmonary atresia with ventricular septal defect, n (%)	**–**	1 (20%)	**–**
Medical history			
Diabetes mellitus, n (%)	–	5 (11.1%)	–
Hypertension, n (%)	–	7 (15.6%)	–
Hyperlipidemia, n (%)	–	3 (6.7%)	–
Medication			
β–Blocker, n (%)	–	3 (6.7%)	–
Calcium channel blockers, n (%)	–	0 (0%)	–
Diuretics, n (%)	–	6 (13.3%)	–
Anticoagulants, n (%)	–	10 (22.2%)	–
ERAs, n (%)	–	24 (53.3%)	–
Prostanoids, n (%)	–	0 (0%)	–
Digoxin, n (%)	–	12 (26.7%)	–
Corticosteroids, n (%)	–	4 (8.9%)	–
PDIs, n (%)	–	26 (57.8%)	–
Right heart catheterization			
RAP, mm Hg	**–**	9 ± 6	–
mPAP, mm Hg	**–**	48 ± 14	–
PCWP, mm Hg	**–**	12 ± 5	–
PVR, Wood units	**–**	11.1 ± 7.0	–
RV systolic pressure, mmHg	**–**	75 ± 25	–
LV function			
LV mass index, g/m^2^	39 ± 8	37 ± 15	0.383
LVEDV index, ml/m^2^	76 ± 11	76 ± 34	0.993
LVESV index, ml/m^2^	32 ± 6	30 ± 21	0.516
LV stroke volume index, ml/m^2^	44 ± 7	47 ± 15	0.399
LVEF, %	59 ± 5	62 ± 9	**0.011**
RV function			
RVEDV index, ml/m^2^	74 ± 13	102 ± 41	**< 0.001**
RVESV index, ml/m^2^	35 ± 8	61 ± 31	**< 0.001**
RV stroke volume index, ml/m^2^	40 ± 6	41 ± 20	0.728
RVEF, %	54 ± 6	42 ± 12	**< 0.001**
RVEDV/LVEDV	0.97 ± 0.10	1.43 ± 0.64	**< 0.001**
RV mass, g	25.6 ± 5.3	39.2 ± 12.4	**< 0.001**
RV/LV mass	0.39 ± 0.06	0.58 ± 0.19	**< 0.001**
TAPSE, mm	19.8 ± 2.8	15.0 ± 3.9	**< 0.001**
RV GLS, %	24.3 ± 3.9	17.9 ± 4.7	**< 0.001**
Right atrial area (end–systole), cm^2^	19.3 ± 4.0	25.3 ± 11.2	**0.001**
PA RAC, %	56 ± 20	25 ± 13	**< 0.001**

Bold values indicated a statistical significance at *P*<0.05

Data are presented as mean ± SD or n (%) or *median (IQR), IQR = 75th percentile–25th percentile. *LV* left ventricle, *RV* right ventricle, *NT-proBNP* N-terminal pro-brain natriuretic peptide, *WHO* World Health Origination, *RAP* right atrial pressure, *mPAP* mean pulmonary artery pressure, *PCWP* pulmonary artery wedge pressure, *PVR* pulmonary vascular resistance, *ERAs* endothelin receptor antagonists, *PDIs* phosphodiesterase inhibitors, *LVEDV* left ventricular end-diastolic volume, *LVESV* left ventricular end-systolic volume, *RVEDV* right ventricular end-diastolic volume, *RVESV* right ventricular end-systolic volume, *TAPSE* tricuspid annular plane systolic excursion, *GLS* global longitudinal strain, *PA RAC* pulmonary artery relative area change, *IQR* interquartile range

### Cardiopulmonary exercise test

PAH group had significantly decreased peak VO_2_, % predicted peak VO_2_, and higher VE/VCO_2_ slope compared with healthy controls (all *P* < 0.001, Table 2). Based on risk score, PAH with intermediate and high risk exhibited significantly reduced peak VO_2_, and % predicted peak VO_2_ compared with PAH with low risk (Additional file 1: Table S2). Based on REVEAL 2.0 score, PAH with intermediate and high risk exhibited significantly reduced % predicted peak VO_2_, and increased VE/VCO_2_ slope compared with PAH with low risk (Additional file 1: Table S2).

**Table 2:** 4D flow and cardiopulmonary exercise test (CPET) parameters in healthy controls and pulmonary arterial hypertension (PAH)

	Healthy control (n = 51)	PAH (n = 45)	*P*^*1,2*^
LV 4D flow			
Direct flow, %	34 (10)	31 (12)	0.474
Retained inflow, %	17 (5)	17 (8)	0.607
Delayed ejection flow, %	17 (5)	16 (7)	0.020
Residual volume, %	33 (6)	36 (10)	0.042
Peak systolic KEI_EDV_, µJ/ml	16.1 (5.1)	19.3 (15.0)	0.012
Average systolic KEI_EDV_, µJ/ml	8.9 (3.1)	11.5 (8.0)	0.025
Peak E–wave KEI_EDV_, µJ/ml	27.0 (12.0)	19.0 (17.4)	**0.002**
RV 4D flow			
Direct flow, %	37 (7)	24 (16)	**< 0.001**
Retained inflow, %	16 (6)	16 (5)	0.956
Delayed ejection flow, %	17 (5)	14 (6)	0.017
Residual volume, %	29 (10)	44 (16)	**< 0.001**
Peak systolic KEI_EDV_, µJ/ml	21.2 (8.5)	19.4 (18.3)	0.521
Average systolic KEI_EDV_, µJ/ml	12.2 (4.5)	10.4 (6.9)	0.070
Peak E–wave KEI_EDV_, µJ/ml	13.9 (7.6)	8.4 (6.3)	**< 0.001**
KE discordance	1.30 (0.53)	0.94 (0.66)	**< 0.001**
Fractional flow ratio	1.24 (0.60)	0.51 (0.40)	**< 0.001**
CPET			
Peak VO_2_, ml/kg/min	22.6 (10.5)	13.2 (4.4)	**< 0.001**
% predicted peak VO_2_, %	87 (33)	48 (23)	**< 0.001**
VE/VCO_2_ slope	27 (4)	41 (10)	**< 0.001**

Bold values indicated a statistical significance at *P*<0.05

Data are presented as median (IQR), IQR = 25th percentile–75th percentile. *LV* left ventricle, *EDV* end-diastolic volume, *KEi*_*EDV*_ kinetic energy normalized to EDV, *RV* right ventricle, *KE discordance* RV/LV average systolic KEI_EDV_, *fractional flow ratio* RV direct flow/RV residual volume, *VO*_*2*_ oxygen uptake, *VE* minute ventilation, *VCO*_*2*_ carbon dioxide output, *IQR* interquartile range. ^1^*P* value from Mann-Whitney U-Test. ^2^Bonferroni significance levels for LV flow parameters, RV flow parameters and CPET parameters are calculated as 0.05/7 = 0.0065; 0.05/9 = 0.0045 and 0.05/3 = 0.017, respectively

### Flow components and kinetic energy

**Fig. 2:**
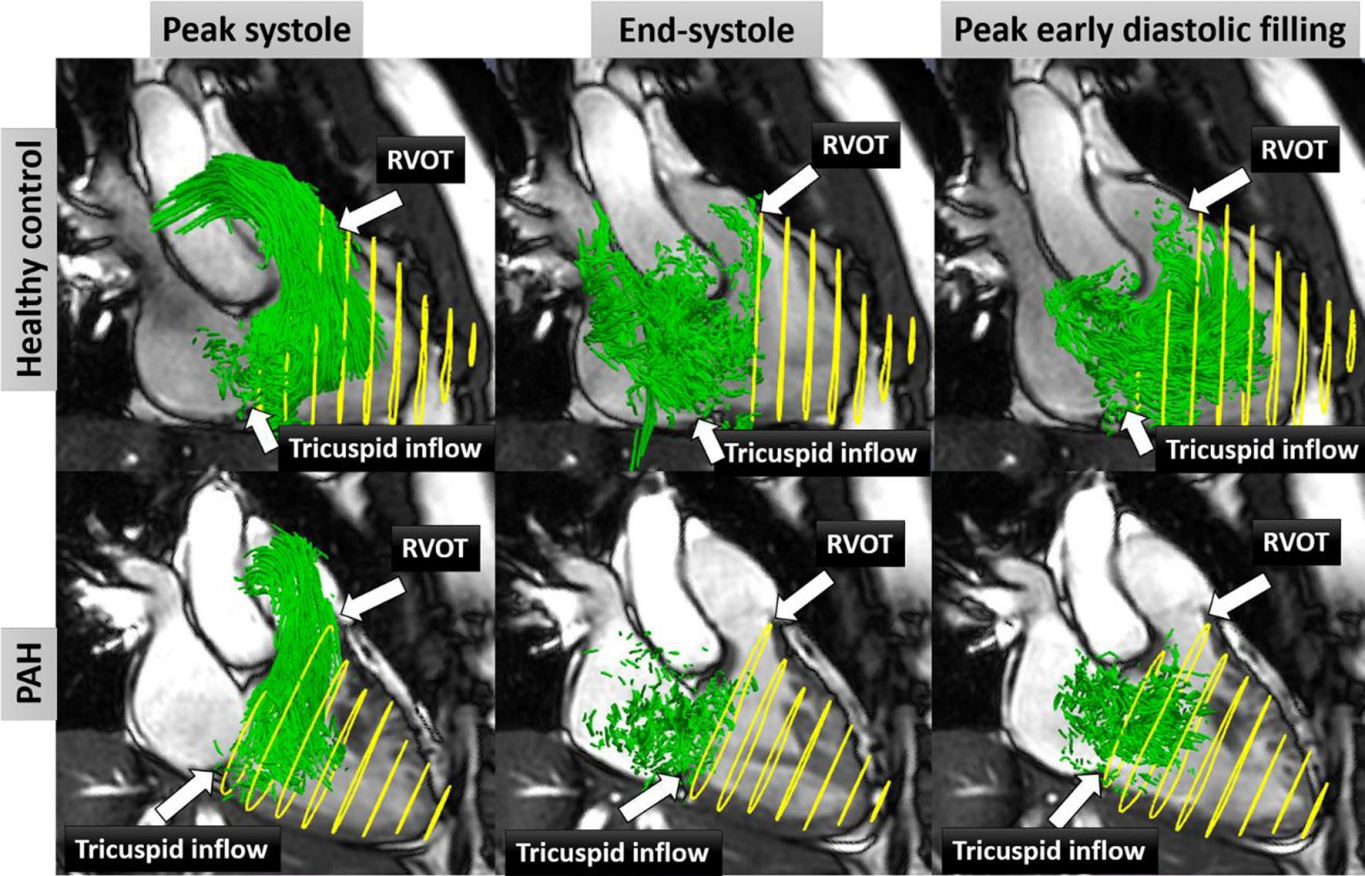
Four-chamber views with right ventricle (RV) direct flow (green) using particle tracing overlayed at peak systole, end-systole and peak early diastolic filling phase in a 48-year-old normal subject (first row), and a 61-year-old pulmonary arterial hypertension (PAH) patient (second row) with RV direct flow 39% and 25%, respectively. Yellow circles denote the RV contours from stacks of short axis views. *RVOT* RV outflow tract

RV direct flow at peak systole, end-systole and peak early diastolic filling for one example each of healthy subject and PAH are shown in Fig. 2. Flow components and KE parameters are tabulated in Table 2. Compared with healthy controls, PAH patients had significantly higher median values of RV residual volume and lower median values of RV direct flow, RV delayed ejection flow, RV peak E-wave KEI_EDV_, KE discordance and fractional flow ratio. Significantly lower median values of RV direct flow and fractional flow ratio, and higher median RV residual volume were observed in PAH patients with intermediate and high risk compared with those with low risk based on both risk score and REVEAL 2.0 score (Additional file 1: Table S2).

RV direct flow was negatively associated with PVR (r=− 0.365, *P* = 0.044); and RV residual volume positively associated with PVR (r = 0.448, *P* = 0.011) and RV systolic pressure (r = 0.399, *P* = 0.032) (Additional file 1: Table S3). There were no significant differences in flow components and KE parameters between PAH patients with and without interventricular mechanical dyssynchrony except median RV peak E-wave KEI_EDV_ (10.6 µJ/ml vs. 7.5 µJ/ml, *P* = 0.042) (Additional file 1: Table S4).

### Association of 4D flow CMR parameters with remodelling index, RV function and CPET outcomes

Both RV direct flow and fractional flow ratio correlated negatively with remodelling index, RA area and VE/VCO_2_ slope, and positively with RVEF, TAPSE, RV GLS, peak VO_2_, and % predicted peak VO_2_ (all *P* < 0.01). RV residual volume correlated negatively with RVEF, TAPSE, RV GLS, peak VO_2_, and % predicted peak VO_2_, and positively with remodelling index, RA area and VE/VCO_2_ slope (all *P* < 0.01) (Additional file 1: Table S5). KE discordance was positively correlated with RVEF, TAPSE, RV GLS, peak VO_2_, and % predicted peak VO_2_, and negatively correlated with VE/VCO_2_ slope (all *P* < 0.05). LV direct flow, LV residual volume and RV KEI_EDV_ parameters were not associated with CPET parameters (Additional file 1: Table S5). A heat map plot was given in Additional file 4: Fig. S2 for easier visualization of these correlations.

**Fig. 3:**
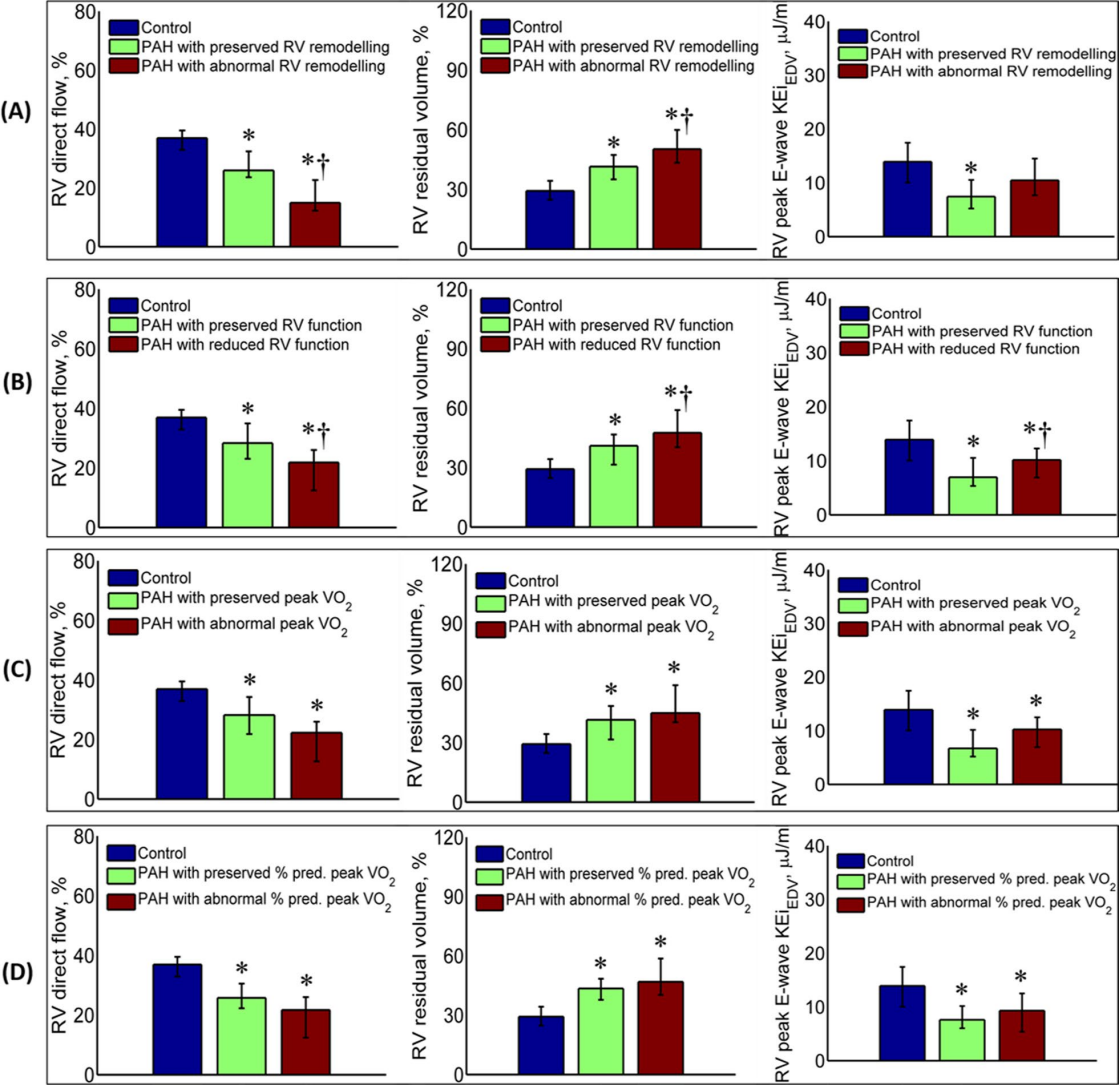
Differences in 4D flow right ventricular (RV) parameters according to RV remodelling; RV function; peak oxygen uptake (VO_2_); and % predicted peak VO_2_. RV direct flow (left), RV residual volume (middle) and RV peak E-wave KEi_EDV_ (right) are presented **A** among healthy controls, PAH with preserved RV remodelling (n = 24) and PAH with abnormal RV remodelling (n = 21); **B** among healthy controls, PAH with preserved RV function (n = 12), and PAH with reduced RV function (n = 33); **C** among healthy controls, PAH with preserved peak VO_2_ (n = 10) and PAH with abnormal peak VO_2_ (n = 35); **D** among healthy controls, PAH with preserved % predicted peak VO_2_ (n = 10) and PAH with abnormal % predicted peak VO_2_ (n = 35). *P < 0.05 compared with healthy controls; †*P* < 0.05 compared with PAH with preserved RV remodelling, and PAH with preserved RV function, respectively. Error bars denote median—25th percentile (lower) and 75th percentile—median (upper)

RV direct flow progressively decreased and RV residual volume increased as RV remodelling index increased (Fig. 3A), and RVEF (Fig. 3B), peak VO_2_ (Fig. 3C) and % predicted peak VO_2_ (Fig. 3D) decreased; moreover, RV direct flow and RV residual volume differed significantly between patients stratified by RV remodelling index (*P* < 0.001 and *P* = 0.002, Fig. 3**(A)**), and RVEF (*P* = 0.007 and *P* = 0.022, Fig. 3B). RV peak E-wave KEI_EDV_ was significantly increased in patients with abnormal RV function compared with patients with preserved RV function (*P* = 0.006, Fig. 3B).

On multivariate linear regression analyses, there were independent positive associations of RV direct flow ($\widehat{\beta }$=0.587, *P* < 0.001), RV GLS ($\widehat{\beta }$=0.596, *P* < 0.001) and RV peak systolic KEI_EDV_ ($\widehat{\beta }$=0.198, *P* = 0.005) with RVEF; independent negative association of RV direct flow ($\widehat{\beta }$=− 0.008, *P* = 0.007) and TAPSE ($\widehat{\beta }$=-0.019, *P* = 0.002) and independent positive association of RA area ($\widehat{\beta }$=0.017, *P* < 0.001) with Ln(RVEDV/LVEDV ratio); and independent positive association of RV direct flow ($\widehat{\beta }$=0.016, *P* < 0.001) and TAPSE ($\widehat{\beta }$=0.035, *P* = 0.001) with Ln(% predicted peak VO_2_) on CPET (Table 3).

**Table 3:** Univariate and multivariable linear regression analyses for determinants of right ventricular (RV) dysfunction, RV remodelling, and exercise capacity in healthy controls and pulmonary arterial hypertension (PAH) patients

	Univariate analysis		Stepwise multivariable analysis	
	**Coefficient (95% CI)**	***P*** **value**	**Coefficient (95% CI)**	***P*** **value**
Determinants of RV function (RVEF)				
RA area, cm^2^	− 0.619 (− 0.839, − 0.398)	< 0.001	–	0.348
TAPSE, mm	1.362 (0.900, 1.823)	< 0.001	**–**	0.514
RV GLS, %	1.430 (1.136, 1.723)	< 0.001	**0.596 (0.281, 0.911)**	**< 0.001**
RV direct flow, %	0.820 (0.686, 0.954)	< 0.001	**0.587 (0.426, 0.748)**	**< 0.001**
RV retained inflow, %	0.076 (− 0.404, 0.556)	0.754	Excluded	
RV delayed ejection flow, %	0.208 (− 0.310, 0.726)	0.428	Excluded	
RV residual volume, %	− 0.649 (− 0.779, − 0.519)	< 0.001	–	0.378
RV peak systolic KEi_EDV_, µJ/ml	0.370 (0.141, 0.600)	0.002	**0.198 (0.061, 0.336)**	**0.005**
RV average systolic KEi_EDV_, µJ/ml	0.747 (0.278, 1.216)	0.002	–	0.802
RV peak E–wave KEi_EDV_, µJ/ml	0.048 (− 0.207, 0.303)	0.710	Excluded	
KE discordance	9.393 (5.136, 13.65)	< 0.001	–	0.846
Fractional flow ratio	11.964 (9.129, 14.80)	< 0.001	–	0.118
R–squared, multivariable				0.698
Determinants of adverse RV remodelling (Ln(RVEDV/LVEDV))*				
RVEF, %	− 0.025 (− 0.033, − 0.017)	< 0.001	–	0.997
RA area, cm^2^	0.042 (0.034, 0.050)	< 0.001	**0.017 (0.011, 0.023)**	**< 0.001**
TAPSE, mm	− 0.051 (− 0.073, − 0.028)	< 0.001	**− 0.019 (− 0.031, − 0.007)**	**0.002**
RV GLS, %	− 0.049 (− 0.065, − 0.033)	< 0.001	–	0.520
RV direct flow, %	− 0.030 (− 0.038, − 0.022)	< 0.001	**− 0.008 (− 0.013, − 0.002)**	**0.007**
RV retained inflow, %	0.006 (− 0.016, 0.028)	0.589	Excluded	
RV delayed ejection flow, %	0.005 (− 0.019, 0.028)	0.703	Excluded	
RV residual volume, %	0.021 (0.014, 0.028)	< 0.001	–	0.385
RV peak systolic KEi_EDV_, µJ/ml	0.003 (− 0.008, 0.014)	0.628	Excluded	
RV average systolic KEi_EDV_, µJ/ml	0.015 (–0.008, 0.037)	0.196	Excluded	
RV peak E–wave KEi_EDV_, µJ/ml	0.008 (− 0.004, 0.019)	0.201	Excluded	
KE discordance	− 0.129 (− 0.342, 0.083)	0.231	Excluded	
Fractional flow ratio	− 0.393 (− 0.544, − 0.241)	< 0.001	**–**	0.096
R–squared, multivariable				0.588
Determinants of exercise capacity (Ln(% predicted peak VO_2_))*				
RVEF, %	0.021 (0.014, 0.028)	< 0.001	–	0.228
RA area, cm^2^	− 0.018 (− 0.028, − 0.008)	0.001	–	0.418
TAPSE, mm	0.055 (0.036, 0.074)	< 0.001	**0.035 (0.014, 0.055)**	**0.001**
RV GLS, %	0.036 (0.021, 0.051)	< 0.001	–	0.096
RV direct flow, %	0.023 (0.015, 0.030)	< 0.001	**0.016 (0.008, 0.024)**	**< 0.001**
RV retained inflow, %	− 0.006 (− 0.025, 0.014)	0.578	Excluded	
RV delayed ejection flow, %	0.019 (− 0.002, 0.040)	0.073	Excluded	
RV residual volume, %	− 0.019 (− 0.025, − 0.012)	< 0.001	**–**	0.209
RV peak systolic KEi_EDV_, µJ/ml	− 0.003 (− 0.013, 0.007)	0.553	Excluded	
RV average systolic KEi_EDV_, µJ/ml	− 0.002 (− 0.023, 0.018)	0.823	Excluded	
RV peak E–wave KEi_EDV_, µJ/ml	− 0.007 (− 0.018, 0.003)	0.164	Excluded	
KE discordance	0.245 (0.060, 0.430)	0.010	–	0.753
Fractional flow ratio	0.331 (0.193, 0.470)	< 0.001	–	0.641
R–squared, multivariable				0.361

Bold values indicated a statistical significance at *P*<0.05

*CI* confidence interval, *RVEF* right ventricular ejection fraction, *RA* right atrial, *TAPSE* tricuspid annular plane systolic excursion, *GLS* global longitudinal strain, *EDV* end-diastolic volume, *KEi*_*EDV*_ kinetic energy normalized to end-diastolic volume, *KE discordance* RV/LV average systolic KEi_EDV_, *fractional flow ratio* RV direct flow/RV residual volume, *RVEDV* right ventricular end-diastolic volume, *LVEDV* left ventricular end-diastolic volume, *LV* left ventricle, *VO*_*2*_ oxygen uptake. *Based on natural log-transformed (Ln) values to ensure normal distributed residual errors

**Fig. 4:**
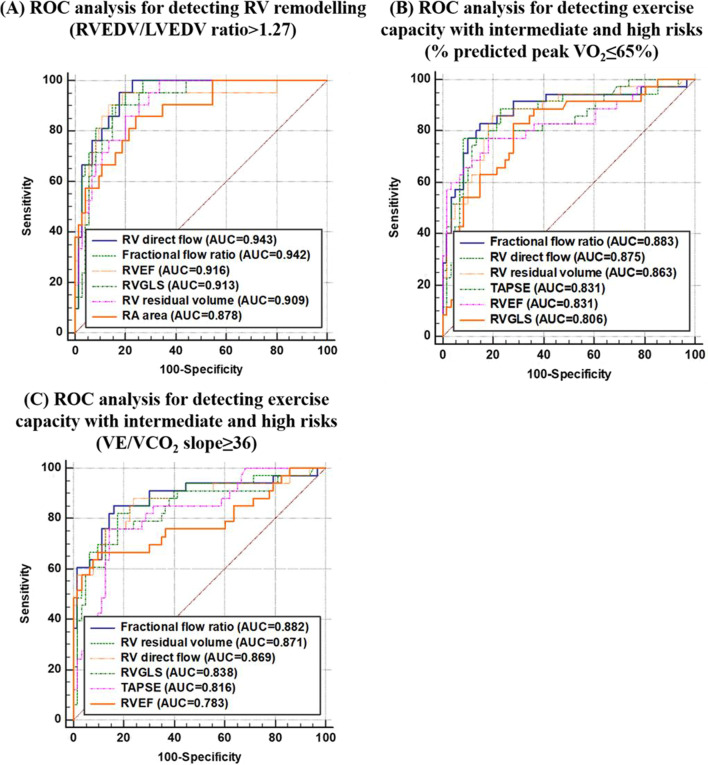
Right ventricular (RV) direct flow, retained inflow, residual volume, fractional flow ratio, RVEF), RV GLS, TAPSE) and right atrial (RA) area to detect **A** RV remodelling (RVEDV)/LVEDV ratio > 1.27), **B** impaired exercise capacity (% predicted peak VO_2_ ≤ 65%), and **C** impaired exercise capacity (VE/VCO_2_ slope ≥ 36). *Fractional flow ratio* RV direct flow/RV residual volume, *RVEF* right ventricular ejection fraction, *GLS* global longitudinal strain, *TAPSE* tricuspid annular plane systolic excursion, *RVEDV* right ventricular end-diastolic volume, LVEDV left ventricular end-diastolic volume, *VO*_*2*_ oxygen uptake, *VE* minute ventilation, *VCO*_*2*_ carbon dioxide output, *AUC* area under curve

**Fig. 5:**
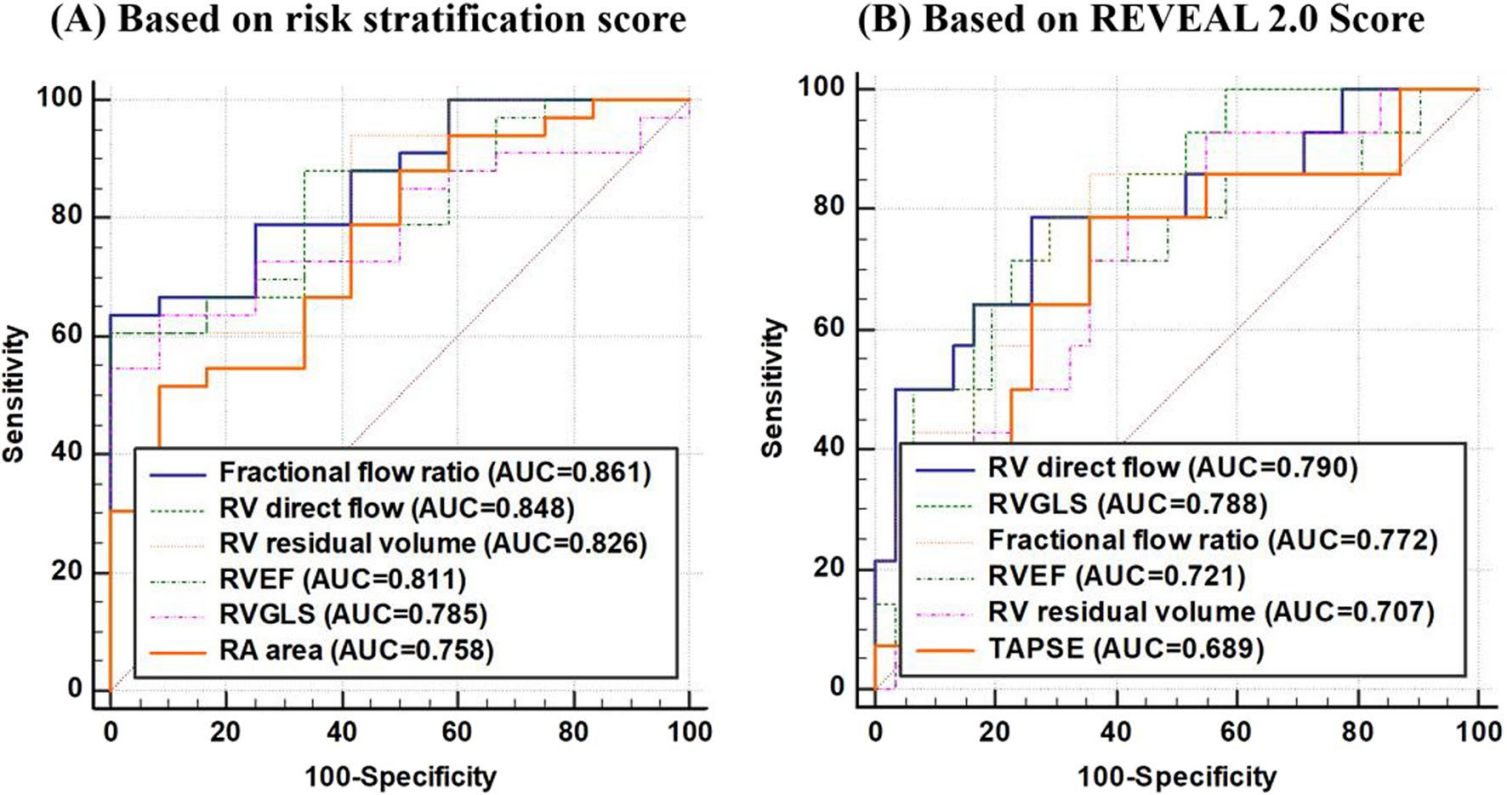
Right ventricular (RV) direct flow, residual volume, fractional flow ratio, RVEF, RV GLS, TAPSE and right atrial (RA) area to detect PAH with intermediate and high risk based on **A** risk stratification score, and **B** REVEAL 2.0 score. *Fractional flow ratio* RV direct flow/RV residual volume, *RVEF* right ventricular ejection fraction, *GLS* global longitudinal strain, *TAPSE* tricuspid annular plane systolic excursion, *PAH* pulmonary arterial hypertension, *REVEAL* Registry to Evaluate Early and Long-Term PAH Disease Management, *AUC* area under curve

**Fig. 6:**
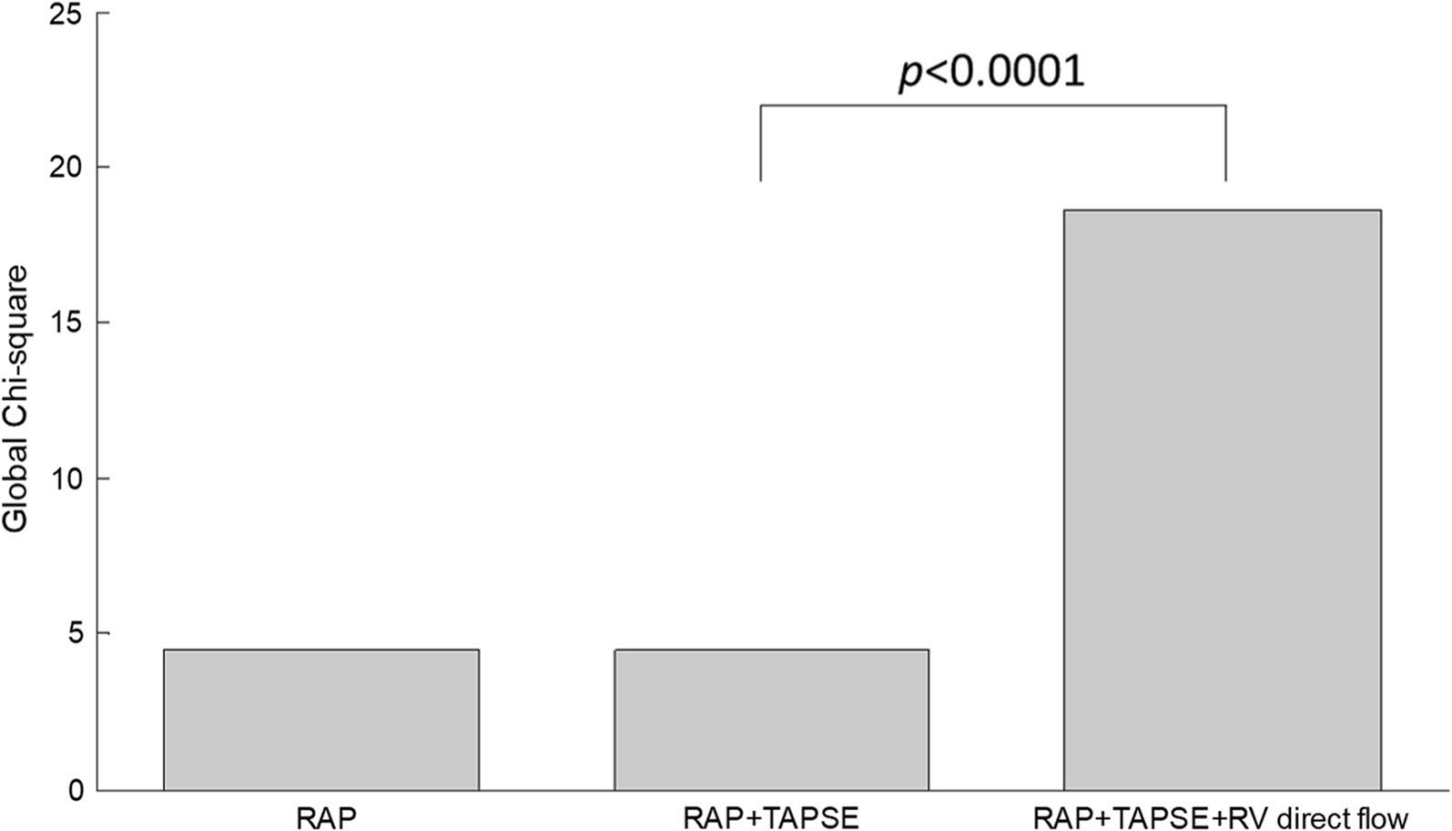
Incremental value of RAP, TAPSE and RV direct flow for discriminating PAH with intermediate and high risk score. Note the marked increase in the cumulative prognostic information provided by RV direct flow over the presence of abnormal TAPSE added to RAP. *RAP* right atrial pressure, *TAPSE* tricuspid annular plane systolic excursion, *RV* right ventricular, *PAH* pulmonary arterial hypertension

On ROC analyses, RV direct flow had higher AUCs than RVEF for adverse RV remodelling, low % predicted peak VO_2_, high VE/VCO_2_ slope, PAH with intermediate and high risk according to risk stratification score and REVEAL 2.0 score (Figs. 4 and 5). The corresponding pairwise (RV direct flow vs. RVEF) DeLong test *P* values were 0.510, 0.263, 0.047, 0.436, and 0.443, respectively, among which only one (for VE/VCO_2_ slope) was significant. On nested binary logistic regression analysis, RV direct flow provided incremental value over RVEF for detecting adverse RV remodelling (*P* = 0.001), exercise intolerance based on % predicted peak VO_2_ ≤ 65% (*P* = 0.004) and VE/VCO_2_ slope ≥ 36 (*P* = 0.001), and PAH with intermediate and high risk based on risk score (*P* = 0.008) (Table 4). In comparison to RAP and TAPSE, RV direct flow was the greatest contributor (incremental ${\chi }^{2}$) to the total model ${\chi }^{2}$ for detecting PAH with intermediate and high risk (Fig. 6).

**Table 4:** Incremental discriminative value of 4D flow parameters

	${\chi }^{2}$for RVEF	Total${\chi }^{2}$for whole model (RVEF + RV direct flow)	*P*
Discriminating adverse RV remodelling	41.36	54.74	**0.001**
Discriminating impaired exercise capacity (% predicted peak VO_2_ ≤ 65%)	40.91	49.15	**0.004**
Discriminating impaired exercise capacity (VE/VCO_2_ slope ≥ 36)	34.85	46.45	**0.001**
Discriminating PAH patients with intermediate and high risk based on risk score	12.32	19.36	**0.008**
Discriminating PAH patients with intermediate and high risk based on REVEAL 2.0 score	7.33	10.07	0.098

Bold values indicated a statistical significance at *P*<0.05

*RV* right ventricular, *RVEF* right ventricular ejection fraction, *VO*_*2*_ oxygen uptake, *VE* minute ventilation, *VCO*_*2*_ carbon dioxide output, *PAH* pulmonary arterial hypertension, *REVEAL* Registry to Evaluate Early and Long-Term PAH Disease Management

*P* denotes the significance of comparison between two Chi-square models.

### Predictors of outcome

During the follow-up period (mean, 34 months; median, 35 [26, 41] months; range, 12–56 months), 9 patients experienced adverse cardiac event (3 deaths; 6 PAH-related hospitalization). However, due to small sample size, 4D flow CMR parameters did not reach statistical significance (Additional file 1: Table S6).

### Reproducibility

Reproducibility results of RV 4D flow CMR parameters for 10 healthy controls and 10 PAH patients are tabulated in Additional file 1: Table S7. Both intra- and interobserver had excellent intraclass correlation coefficients (all ≥ 0.94, *P* < 0.001). Mean intra- and interobserver difference measurements were small with good limits of agreement (Additional file 1: Table S7). Bland-Altman plots of intra- and interobserver measurements are shown in Additional file 4: Fig. S3. Coefficients of variation for interobserver reproducibility were 2.4%, 8.7%, 7.8%, 2.2% for RV direct flow, retained inflow, delayed ejection flow and residual volume, respectively; and for intraobserver reproducibility 2.3%, 7.8%, 6.0% and 2.1%, respectively. For RV peak systolic, average systolic and peak E-wave KEI_EDV_, the respective interobserver coefficients of variation were 4.9%, 4.4% and 4.6% and intraobserver coefficients of variation 2.6%, 3.5% and 2.0%.

## Discussion

In this study, we examined associations of 4D flow CMR-derived LV and RV flow components and KE with RV remodelling, RV functional and CPET outcomes. RV direct flow was significantly decreased in PAH patients compared with healthy controls and was demonstrated on nested binary logistic regression analysis to provide incremental value over RVEF for discriminating adverse RV remodelling, impaired exercise capacity, and PAH with intermediate and high risk based on risk score (Table 4). While *post hoc* pairwise comparisons of AUCs between RV direct flow and RVEF using the DeLong test were mostly nonsignificant, the study conclusion was not necessarily negated. The DeLong test suffered loss of power in numerical simulations, might be insensitive and could potentially miss out significant risk predictors [35]. Further, using both predictive modelling and ROC comparison constituted double-testing of the null hypothesis, which inherently reduced power, and single tests for assessing the incremental predictive accuracy of new markers might be more appropriate [36]. Hence, we believe our overall results support the incremental value of 4D flow CMR over standard cine CMR in the assessment of PAH patients.

### Novelty of the study

To the best of our knowledge, this paper is the largest 4D flow CMR analysis for RV flow components and KE parameters in patients with PAH and the first to report on the association of these parameters with CPET outcome. Several prior 4D flow CMR research studies have reported perturbations of PA hemodynamics in PAH patients [7–12], but only few have focused on intracardiac flow analysis in terms of RV KE work density [13], vorticity [37] and RV flow components [14]. In 10 functional class I/II PAH patients and 9 healthy controls, Han et al. [13] studied RV KE work density defined as the minimum kinetic work of the heart required per stroke volume which included the contribution of RV work. The estimated systolic RV work density in our study is significantly greater (52.5 vs. 40.9 µJ/ml, *P* = 0.003) in PAH patients compared with controls, which is in agreement with the findings [13]. It is noted that our study has normalized RV KE to end-diastolic volume which precludes direct comparison. In addition, we have associated RV KE and flow components with CPET and found its added value of prognosis in PAH. In [37], Fenster et al. investigated the associations of 4D flow-derived intracardiac vorticity and RV diastolic dysfunction using echocardiographic indexes in 13 PAH patients and 10 controls, and RV diastolic dysfunction was associated with alterations with E-wave and A-wave vorticities. More recently, Wang et al. [14] analyzed the RV flow components using CVI^42^ in 30 PAH patients and 14 healthy controls, and reported significantly reduced RV direct flow and increased RV residual volume; in addition, RV direction flow was negatively correlated with PVR in PAH patients. The findings of RV flow components are in agreement with our results, while no RV KE parameters were analyzed in [14]. These prior studies enrolled modest cohorts and, more importantly, unlike us, they did not investigate exercise performance, which is an important prognostic determinant in PAH.

Our study investigates associations of 4D flow-derived RV flow components and KE with a clinically relevant outcome like exercise tolerance in PAH patients. RV direct flow component was found to correlate with RV remodelling, function and CPET outcomes and demonstrated better associations with indexes of RV remodelling, exercise intolerance, and risk scores than standard CMR volumes and function parameters. This suggests that 4D flow CMR-derived measurements may be useful for monitoring RV dysfunction and risk prognostication in PAH patients.

### Advantages of 4D flow CMR

4D flow CMR provides unprecedented capabilities for comprehensive analysis of complex blood flow patterns and has been applied in various cardiovascular diseases [38]. Standardization of 4D flow CMR in CHD has been recommended for its acquisition, reconstruction and postprocessing [6]. With advances in technology and big-data processing, 4D flow CMR requires shorter time for acquisition (< 10 min) and pre- and postprocessing. For the calculation of 4D flow parameters in this paper, segmentation of ventricular contours based on artificial intelligence in stacks of cine short axis images for whole cardiac cycles using MASS consumes similar time compared with manual segmentation of the end-diastolic and end-systolic phases for the volumetric function analysis. In this study, the value proposition of 4D flow CMR is that it can potentially combine standard imaging parameters with new physiological insights that have prognostic significance. The preliminary results here will have to be confirmed in larger cohorts before we can decide whether 4D flow CMR parameters can replace some of the other factors or to be added to guidelines for better early diagnosis and prognostication of PAH.

With the availability of 4D flow analysis software, e.g., MASS and CVI^42^, 4D flow CMR has garnered attention in the community, especially for its accurate assessment of valve regurgitation, which can be used to guide the timing of surgical intervention and assess the post-surgery result. With advances in the acquisition and AI analysis of 4D flow CMR, future updated guidelines may consider to include 4D flow CMR parameters for baseline and follow-up assessment.

### CPET as a surrogate marker of clinical outcome

PAH is a rare disorder with unfavourable prognosis, and its survival remains poor despite advances in the therapeutic management and implementation of specific PAH-oriented therapy. Therefore, the use of surrogate markers is necessary given the rarity of PAH and dearth of large trials, especially in drug discovery. For want of an expedient alternative, 6MWT is widely used to quantify exercise capacity in PAH patients and is the clinical reference standard for assessing disease progression and response to treatment [39]. The 6MWT has been shown to be associated with long-term outcomes in PAH patients from the randomized SERAPHIN trial [40], where patients with a 6MWT > 400 m have a reduced risk of PAH-related death or hospitalization. However, improvement in 6MWT does not necessarily reflect benefit in clinical outcomes [41] and the change from baseline in 6MWT is a weak surrogate of clinical outcomes [42]. Unlike 6MWT, CPET provides a comprehensive pathophysiological evaluation of patients’ exercise limitation and dyspnoea, which are the main and early symptoms of the disease [16]. Moreover, CPET does provide good prognostic insights in experienced hands. In 226 patients with idiopathic or familiar PAH during follow-up (1508 ± 1070 days), the combination of peak VO_2_ and PVR provided accurate risk stratification and complementary prognostic information [43]. In another study that investigated the added value of CPET in the follow-up of idiopathic, heritable and drug-induced PAH patients in both derivation cohort (n = 80) and validation cohort (n = 80) [44], the authors showed that the combination of baseline peak VO_2_ from CPET and change in cardiac index during follow-up is important for the prognostication of PAH patients with low risk. Moreover, a recent paper explored the risk thresholds and predictive capacity of CPET on 5-year mortality in idiopathic PAH patients (n = 210, median follow-up: 34 months) [45]. From the multivariable Cox regression analysis, three CPET variables were independently predictive of mortality with one of them being peak VO_2_.

A major strength of our study is the inclusion of CPET, which through specifically using peak VO_2_ based parameters as a prognostic tool for follow-up, offers a more granular assessment of functional status and RV function than the 6MWT in PAH patients [16]. In this study, for the first time we were able to dissect the associations between 4D flow CMR parameters and detailed CPET outcomes. RV direct flow—but none of the other 4D flow or standard CMR volume and function parameters—was independently associated with % predicted peak VO_2_, which supports its use as a surrogate marker of disease progression, therapeutic response in the clinic and in trials, and potentially, clinical prognosis.

## Potential clinical value of intracardiac flow component and kinetic energy assessment

The RV mass [46] and PA relative area change [28, 29] have been reported and shown to be strong prognostic markers in other pulmonary hypertension studies. Compared with those values, our RV mass was significantly smaller than those reported in [46] for 26 PAH as trabecular muscle was included in their RV mass calculation; our pulmonary artery relative area change was comparable with those in [28] for 70 PAH patients (25 ± 13 vs. 20 ± 10%) and larger than those in [29] for 115 PAH patients (25 ± 13 vs. 8.1 ± 6.5%). RV function has been shown to be the single most important prognostic determinant of survival in PAH [47], and several knowledge gaps and research opportunities have been identified by the American Thoracic Society Research Statement [48], with one being the assessment of RV diastolic function. The systemic review by Barker et al. [49] indicated that direct flow through the RV by 4D flow CMR was of high importance when performing such assessment. Therefore, this novel, non-invasive measurement from 4D flow CMR could provide incremental information for assessment of RV diastolic dysfunction and may be considered as prognostic marker which needs to be confirmed with diastolic pressure-volume curves from invasive right heart catheterization.

RVEDV/LVEDV ratio, which we termed remodelling index, when being added to indexed RVEDV, can increase the sensitivity to detect RV enlargement in PAH patients [50]. Given the significant association between RV direct flow and remodelling index (r=-0.624, Additional file 1: Table S5), the value of RV direct flow for detecting early RV dilatation in PAH needs to be investigated in future studies. Another study [51] showed that RVEDV/LVEDV ratio was associated with increased all-cause mortality in patients with PAH, we believe that RV flow component will shed light on the prognostication of PAH based on the its superior discrimination for PAH patients with intermediate and high risk (Fig. 5) and the Cox regression analysis results (Additional file 1: Table S6). Recent guideline recommends a multidimensional approach to risk assessment in PAH. Patients classified as intermediate and high risk have an estimated life expectancy of one year [2]. Several variables determined by CPET provide prognostic information and exercise capacity as quantified by peak VO_2_, which is the most widely used measure for therapeutic decision making [43]. We found that median RV direct flow was significantly reduced in PAH with intermediate and high risk based on risk score (18% vs. 30%) and REVEAL 2.0 score (13 vs. 25%) (Additional file 1: Table S2). In addition, our study demonstrated that RV direct flow is a more sensitive parameter of peak VO_2_ than conventional RV volume and function measurements. Future incorporation of these advanced imaging parameters may help to redefine and improve prognostic capability for those PAH patients at greatest risk of complications.

### Role of CPET in PAH

Our PAH cohort comprises Group 1 pulmonary hypertension patients with heterogeneous disease etiologies, including idiopathic PAH, and PAH associated with CHD and different types of CTD. The small size of our PAH cohort precluded meaningful separate analyses of CPET outcomes by the different etiologies. There are limited studies in the literature that compared CPET outcomes in PAH patients with different etiologies. Bellan et al. [52] studied 8 and 112 CTD patients with and without PAH, respectively, and observed in the former group significantly lower peak VO_2_ and steeper VE/VCO_2_ slope, which yielded the best AUCs of 0.845 and 0.888, respectively, for diagnosis of PAH in CTD patients among various CPET parameters studied. Moreover, the authors found peak VO_2_ and VE/VCO_2_ slope to be comparable between the 8 CTD patients with PAH and 11 other patients with pulmonary hypertension of diverse etiologies [52], which lends support to the interpretability of our study results. Similarly, Zhang et al. [53] observed no significant differences in peak VO_2_ and VE/VCO_2_ slope between 93 CTD with PAH and 93 age-, gender-, body mass index- and BSA-matched patients with idiopathic PAH, suggesting again that these CPET parameters probably reflect more of the PAH status than the underlying CTD etiology. With regard to CHD, Righini et al. [54] compared 57 and 110 PAH patients with and without CHD, respectively, and found significantly lower peak VO_2_ and higher VE/VCO_2_ slope in the former group. However, the latter group comprised mainly Group 4 pulmonary hypertension subjects [54], who may not provide the best controls for PAH patients. Among our PAH subjects, we believe that intragroup similarities, i.e., common pathophysiological and hemodynamic underpinnings, constitute a major determinant of CPET outcomes, specifically peak VO_2_ and VE/VCO_2_ slope, which are primary outcomes in our study, more than differences attributable to individual etiologies.

### Study implication

4D flow CMR measures velocities simultaneously in three directions over the entire cardiac cycle, allowing comprehensive flow assessment in any direction in the volume of interest. Through use of visualization tools, 4D flow CMR has universalized the representation of complex intracardiac flow patterns like vortex formation and turbulent flow in diverse myocardial and valvular pathologies. It enables quantitation of flow components—direct flow, retained inflow, delayed ejection flow, and residual volume—as well as phasic KE, which may show up subtle myocardial dysfunction. In [55], RV dysfunction was demonstrated by impaired RV direct flow and KE but not standard CMR measures in 22 subjects with primary LV disease. In the current study of 45 PAH subjects, RV direct flow was significantly reduced compared with healthy controls (24 vs. 37%, *P* < 0.001) and was an independent marker for RV remodelling, function and exercise capacity based on % predicted peak VO_2_. The significant correlation of RV direct flow with exercise capacity suggests potential prognostic value that warrants further longitudinal investigation.

### Study limitations

Our study has several limitations. First, this is a cross-sectional observational study, and causality in relationships cannot be inferred. Second, the study included only patients with PAH. Whether the findings can be extrapolated to other pulmonary hypertension groups is unknown. More studies involving all categories of clinical pulmonary hypertension would be needed to confirm the wider applicability of 4D flow CMR. Third, 60% of PAH patients in the study were in WHO functional class I. We did include a significant minority of patients at intermediate and higher risk as assessed by objective clinical, RHC, 6MWT and CPET parameters by [16]. Further studies on larger cohorts involving more symptomatic patients are needed to confirm our observations on the associations of intracardiac 4D flow CMR components with functional status. Another limitation of this study was the inclusion of Group 1 pulmonary hypertension patients with heterogeneous etiologies. While the small study sample precluded separate analyses of CPET outcomes by etiology, the intragroup similarities among PAH patients―including common pathophysiological, hemodynamic, prognostic, and management features, as well as, in some studies, CPET outcomes [52, 53] should provide the basis and guidance for interpreting our overall results. CMR as a powerful marker in PAH has been confirmed by the systematic review and meta-analysis in [56], and RV function and volumes can predict mortality and clinical worsening. Given the association of RV direct flow with RV function and volumes, a longitudinal study with clinical outcomes would be needed to confirm the prognostic significance of 4D flow CMR parameters. Clinical outcomes associated with PAH patients were analyzed using the Cox proportional-hazards regression analysis, although statistical significance was not achieved due to the small sample size (n = 9, 20%; Additional file 1: Table S6). Future large multi-centre longitudinal study with clinical outcomes is needed to assess the prognostic value of these new 4D flow CMR parameters on clinical outcomes.

## Conclusion

The RV direct flow component identified abnormalities of RV function in PAH patients and was associated with RV remodelling and exercise test outcomes in these patients. Hence, it may serve as a useful surrogate outcome for follow-up of disease progression and therapeutic response in PAH with application in both clinical and research settings.

## Supplementary Information

**Additional file 1: Table S1.** Acquisition parameters of 4D flow CMR imaging in two centres. **Table S2.** Comparison of RV function, 4D flow and cardiopulmonary exercise test (CPET) parameters between pulmonary arterial hypertension (PAH) with low risk, and intermediate and high risk based on risk stratification score and REVEAL 2.0 score. **Table S3.** Correlations between PVR, RV systolic pressure and 4D flow parameters in pulmonary arterial hypertension (PAH). **Table S4.** 4D flow parameters in pulmonary arterial hypertension (PAH) without interventricular mechanical dyssynchrony versus with interventricular dyssynchrony. **Table S5.** Correlation coefficient R of 4D flow parameters and right ventricular (RV) remodelling, RV function, six-minute walk test (6MWT) and cardiopulmonary exercise test (CPET) parameters in healthy controls and pulmonary arterial hypertension (PAH). **Table S6.** Predictors of the prospectively identified events using Cox regression. **Table S7.** Intra- and interobserver agreement of right ventricular (RV) flow components and kinetic energy parameters.

**Additional file 2.** Methods for 4D flow analysis.

**Additional file 3.** Movies showing four-chamber views with right ventricle (RV) four flow components using particle tracing in a 48-year-old healthy subject, and a 61-year-old pulmonary arterial hypertension (PAH) patient. Yellow circles denote the RV contours from stacks of short axis views. Color legend: green (RV direct flow), yellow (RV retained inflow), blue (RV delayed ejection flow), red (RV residual volume).

**Additional file 4: Figure S1. **Difference in time to maximal displacement between right ventricle (RV) free wall and left ventricle (LV) lateral wall (Time difference = RV-LV) for healthy control (left) and PAH (right). **Figure S2. **Heat map for the correlation coefficient R of 4D flow parameters and right ventricular (RV) remodelling, RV function, 6MWT  and cardiopulmonary exercise test (CPET) parameters in healthy controls and pulmonary arterial hypertension (PAH). **Figure S3.** Bland-Altman analysis of right ventricular (RV) 4D flow measurements for (**A**) intraobserver; (**B)** interobserver for RV direct flow (first row, left), RV retained inflow (first row, right), RV delayed ejection flow (second row, left), RV residual volume (second row, right), RV peak systolic KEi_EDV_ (third row, left), RV systolic KEi_EDV_ (third row, right), and RV peak E-wave KEi_EDV_ (last row).

## Data Availability

The datasets used and/or analysed during the current study are available from the corresponding author on reasonable request.

## Abbreviations

4D: Four-dimensional
6MWT: Six-minute walk test
AUC: Area under ROC curve
CHD: Congenital heart disease
CI: Confidence interval
CMR: Cardiovascular magnetic resonance
CPET: Cardiopulmonary exercise testing
CTD: Connective tissue disease
CV: Coefficients of variation
EDV: End-diastolic volume
EF: Ejection fraction
ESV: End-systolic volume
GLS: Global longitudinal strain
ICC: intraclass correlation coefficient
IQR: interquartile range
KE: Kinetic energy
KEI_EDV_: KE normalized to end-diastolic volume
LV: Left ventricle/left ventricular
LVEDV: left ventricular end-diastolic volume
LVEF: left ventricular ejection fraction
LVESV: left ventricular end-systolic volume
mPAP: Mean pulmonary artery pressure
PA: Pulmonary artery
PAH: Pulmonary arterial hypertension
PCWP: Pulmonary artery wedge pressure
PVR: Pulmonary vascular resistance
RA: Right atrium/right atrial
RAC: Relative area change
RAP: Right atrial pressure
REVEAL: Registry to Evaluate Early and Long-Term PAH Disease Management
RHC: Right heart catheterization
ROC: Receiver operating characteristic
RV: Right ventricle/right ventricular
RVDD: Right ventricular diastolic dysfunction
RVEDV: Right ventricular end-diastolic volume
RVEF: Right ventricular ejection fraction
RVESV: Right ventricular end-systolic volume
RVOT: Right ventricular outflow tract
RVSP: RV systolic pressure
SD: Standard deviation
SENSE: Sensitivity encoding
SV: Stroke volume
TAPSE: Tricuspid annular plane systolic excursion
VCO_2_: Carbon dioxide production
VE: Minute ventilation
VO_2_: Oxygen uptake
WHO: World Health Organization
