# Supplementary material for: Right ventricular energetic biomarkers from 4D Flow CMR are associated with exertional capacity in pulmonary arterial hypertension
Source: J Cardiovasc Magn Reson. 2022 Dec 1;24:61. doi: 10.1186/s12968-022-00896-8 (PMC9714144; doi:10.1186/s12968-022-00896-8)
Supplement: Supplementary file 1 — Additional file 1: Table S1. Acquisition parameters of 4D flow CMR imaging in two centres. Table S2. Comparison of RV function, 4D flow and cardiopulmonary exercise test (CPET) parameters between pulmonary arterial hypertension (PAH) with low risk, and intermediate and high risk based on risk stratification score and REVEAL 2.0 score. Table S3. Correlations between PVR, RV systolic pressure and 4D flow parameters in pulmonary arterial hypertension (PAH). Table S4. 4D flow parameters in pulmonary arterial hypertension (PAH) without interventricular mechanical dyssynchrony versus with interventricular dyssynchrony. Table S5. Correlation coefficient R of 4D flow parameters and right ventricular (RV) remodelling, RV function, six-minute walk test (6MWT) and cardiopulmonary exercise test (CPET) parameters in healthy controls and pulmonary arterial hypertension (PAH). Table S6. Predictors of the prospectively identified events using Cox regression. Table S7. Intra- and interobserver agreement of right ventricular (RV) flow components and kinetic energy parameters. [file 12968_2022_896_MOESM1_ESM.docx]

**Table S1** Acquisition parameters of 4D flow cardiovascular magnetic resonance (CMR) imaging in two centres.

| Vendor | Philips | Siemens |
| --- | --- | --- |
| Magnetic field strength | 3T | 1.5T |
| Pulse sequence | Spoiled gradient echo | Spoiled gradient echo |
| Acceleration method | EPI factor 5 and SENSE factor 2 | GRAPPA factor 2 |
| Field of view (mm^2^) | 340 × 340 | 340 × 236.8 |
| Slice orientation | Coronal | Sagittal |
| Acquired voxel size (mm^3^) | 3.0 × 3.0 × 3.0 | 3.0 × 3.0 × 3.0 |
| TR/TE (ms) | 6.4-12.0/3.3-4.0 | 40.56/2.94 |
| Flip angle (°) | 10 | 9 |
| Cardiac phases | 30 | 27-33  (Depending on RR interval) |
| VENC (cm/s) | 150 (maximum 220) | 220 |
| Cardiac gating | Retrospective ECG gating | Prospective ECG gating |
| Respiratory motion | Free breathing | Free breathing |
| Scan time (min) | 5 - 10 | 20 |

*4D* four-dimensional, *ECG* electrocardiogram, *EPI* echo-planar imaging, *GRAPPA* generalized autocalibrating partially parallel acquisition, *kat-ARC* k-adaptive-t autocalibrating reconstruction for Cartesian sampling, *SENSE* sensitivity encoding, *TR* repetition time, *TE* echo time, *VENC* velocity encoding.

**Table S2** Comparison of right ventricular (RV) function, 4D flow and cardiopulmonary exercise test (CPET) parameters between pulmonary arterial hypertension (PAH) with low risk, and intermediate and high risk based on risk score and REVEAL 2.0 score.

|  | **Risk score** | | | **REVEAL 2.0 score** | | |
| --- | --- | --- | --- | --- | --- | --- |
|  | **Low risk**  **(n=12)** | **Intermediate and high risk (n=33)** | ***P*** | **Low risk**  **(n=31)** | **Intermediate and high risk (n=14)** | ***P*** |
| NT-proBNP, pg/mL* | 124 (128) | 359 (922) | **0.003** | 149 (174) | 444 (1738) | **<0.001** |
| **RV function** | | | |  | | |
| RVEDV index, ml/m^2^ | 86 ± 25 | 107 ± 44 | 0.172 | 94 ± 28 | 117 ± 58 | 0.182 |
| RVESV index, ml/m^2^ | 43 ± 15 | 68 ± 33 | **0.009** | 54 ± 26 | 77 ± 38 | **0.023** |
| RV SV index, ml/m^2^ | 43 ± 12 | 40 ± 22 | 0.093 | 41 ± 10 | 41 ± 32 | 0.985 |
| RVEF, % | 50 ± 6 | 38 ± 12 | **0.001** | 45 ± 10 | 35 ± 13 | **0.006** |
| RVEDV/LVEDV | 1.17 ± 0.34 | 1.53 ± 0.69 | 0.156 | 1.28 ± 0.44 | 1.76 ± 0.86 | 0.065 |
| TAPSE, mm | 16.0 ± 4.0 | 14.6 ± 3.9 | 0.454 | 15.8 ± 3.6 | 13.3 ± 4.1 | **0.048** |
| RV GLS, % | 20.9 ± 2.9 | 16.7 ± 4.7 | **0.003** | 19.2 ± 4.4 | 14.7 ± 3.7 | **0.002** |
| Right atrial area (end-systole), cm^2^ | 18.9 ± 5.3 | 27.7 ± 11.9 | **0.008** | 23.5 ± 8.9 | 29.3 ± 14.8 | 0.190 |
| **LV 4D flow*** | | | |  | | |
| Direct flow, % | 34 (15) | 31 (10) | 0.257 | 31 (11) | 33 (15) | 0.787 |
| Retained inflow, % | 17 (8) | 17 (7) | 0.603 | 17 (8) | 17 (6) | 0.961 |
| Delayed ejection flow, % | 16 (8) | 16 (7) | 0.810 | 16 (8) | 16 (8) | 0.864 |
| Residual volume, % | 31 (15) | 36 (9) | 0.315 | 36 (10) | 33 (10) | 0.624 |
| Peak systolic KEi_EDV_, µJ/ml | 19.9 (19.4) | 19.3 (15.0) | 0.830 | 19.3 (14.6) | 20.0 (17.5) | 0.650 |
| Average systolic KEi_EDV_, µJ/ml | 10.9 (11.5) | 11.5 (6.6) | 0.929 | 10.9 (8.5) | 11.7 (7.5) | 0.540 |
| Peak E-wave KEi_EDV_, µJ/ml | 18.4 (17.8) | 19.0 (17.8) | 0.752 | 20.0 (16.7) | 13.3 (23.9) | 0.447 |
| **RV 4D flow*** | | | |  | | |
| Direct flow, % | 30 (12) | 18 (13) | **<0.001** | 25 (13) | 13 (11) | **0.002** |
| Retained inflow, % | 15 (6) | 16 (4) | 0.551 | 16 (6) | 16 (1) | 0.633 |
| Delayed ejection flow, % | 15 (7) | 13 (6) | 0.454 | 13 (6) | 14 (8) | 0.750 |
| Residual volume, % | 38 (13) | 49 (18) | **0.001** | 43 (12) | 50 (17) | **0.027** |
| Peak systolic KEi_EDV_, µJ/ml | 20.7 (19.7) | 18.9 (17.9) | 0.621 | 19.3 (14.8) | 18.5 (22.1) | 0.405 |
| Average systolic KEi_EDV_, µJ/ml | 10.7 (8.2) | 10.4 (6.7) | 0.929 | 11.1 (7.2) | 7.9 (7.8) | 0.607 |
| Peak E-wave KEi_EDV_, µJ/ml | 7.8 (4.1) | 10.3 (10.5) | 0.236 | 8.2 (5.0) | 9.4 (14.8) | 0.633 |
| KE discordance | 0.90 (0.59) | 0.94 (0.71) | 0.849 | 0.96 (0.60) | 0.71 (1.00) | 0.194 |
| Fractional flow ratio | 0.73 (0.56) | 0.38 (0.37) | **<0.001** | 0.59 (0.47) | 0.29 (0.29) | **0.004** |
| **CPET*** | | | |  | | |
| Peak VO_2_, ml/kg/min | 15.2 (4.7) | 11.4 (4.5) | **<0.001** | 13.9 (5.6) | 12.2 (4.7) | 0.274 |
| % predicted peak VO_2_, % | 67 (36) | 46 (15) | **0.001** | 52 (22) | 44 (12) | **0.011** |
| VE/VCO_2_ slope | 38 (15) | 41 (12) | 0.098 | 39 (11) | 45 (13) | **0.038** |

Data are presented as mean±SD or *median (IQR), IQR=75^th^ percentile–25^th^ percentile. *REVEAL* Registry to Evaluate Early and Long-Term PAH Disease Management, *NT-proBNP* N-terminal pro-brain natriuretic peptide, *RV* right ventricle, *RVEDV* end-diastolic volume, RVEF, right ventricular ejection fraction, RVESV end-systolic volume, *SV* stroke volume, *TAPSE* tricuspid annular plane systolic excursion, *GLS* global longitudinal strain, *LV* left ventricle, *KEi_EDV_* kinetic energy normalized to EDV, *KE discordance* RV/LV average systolic KEi_EDV_, *fractional flow ratio* RV direct flow/RV residual volume, *VO_2_* oxygen uptake, *VE* minute ventilation, *VCO_2_* carbon dioxide output.

**Table S3** Correlations between pulmonary vascular resistance (PVR), RVRV systolic pressure and 4D flow parameters in pulmonary arterial hypertension (PAH)

| **4D flow parameters** | **PVR, Wood units*** | ***P* value for correlation with PVR** | **RV systolic pressure, mmHg**† | ***P* value for correlation with RVSP** |
| --- | --- | --- | --- | --- |
| RV direct flow, % | **-0.365** | **0.044** | -0.358 | 0.056 |
| RV retained inflow, % | -0.123 | 0.511 | -0.207 | 0.280 |
| RV delayed ejection flow, % | -0.210 | 0.256 | -0.022 | 0.910 |
| RV residual volume, % | **0.448** | **0.011** | **0.399** | **0.032** |
| RV peak systolic KEi_EDV_, µJ/ml | 0.027 | 0.887 | 0.103 | 0.596 |
| RV average systolic KEi_EDV_, µJ/ml | 0.076 | 0.685 | 0.192 | 0.318 |
| RV peak E-wave KEi_EDV_, µJ/ml | -0.046 | 0.807 | 0.020 | 0.919 |
| KE discordance | 0.122 | 0.513 | 0.085 | 0.660 |
| Fractional flow ratio | -0.279 | 0.129 | -0.307 | 0.099 |

*PVR* pulmonary vascular resistance, *RV* right ventricular, *KEi_EDV_* kinetic energy normalized to end-diastolic volume, *KE discordance* RV/LV average systolic KEi_EDV_, *fractional flow ratio* RV direct flow/RV residual volume, *LV* left ventricle. *PVR measurements were available in 31 PAH patients; †RV systolic pressure measurements were available in 29 PAH patients.

**Table S4** 4D flow parameters in pulmonary arterial hypertension (PAH) without interventricular mechanical dyssynchrony versus with interventricular dyssynchrony.

| **4D flow parameters** | **PAH without interventricular mechanical dyssynchrony (n=28)*** | **PAH with interventricular mechanical dyssynchrony (n=17)*** | ***P*** † |
| --- | --- | --- | --- |
| RV direct flow, % | 25 (15) | 22 (15) | 0.399 |
| RV retained inflow, % | 16 (6) | 16 (3) | 0.520 |
| RV delayed ejection flow, % | 13 (6) | 15 (6) | 0.251 |
| RV residual volume, % | 45 (13) | 44 (18) | 0.590 |
| RV peak systolic KEi_EDV_, µJ/ml | 18.8 (16.6) | 25.6 (21.6) | 0.468 |
| RV average systolic KEi_EDV_, µJ/ml | 9.8 (6.8) | 11.9 (7.9) | 0.374 |
| RV peak E-wave KEi_EDV_, µJ/ml | 7.5 (5.6) | 10.6 (8.2) | **0.042** |
| KE discordance | 0.97 (0.69) | 0.82 (0.65) | 0.656 |
| Fractional flow ratio | 0.53 (0.41) | 0.43 (0.42) | 0.386 |

Data are presented as median (IQR), IQR=75^th^ percentile–25^th^ percentile.

*IQR* interquartile range, *KE discordance* RV/LV average systolic KEi_EDV_, *fractional flow ratio* RV direct flow/RV residual volume, *KEi_EDV_* kinetic energy normalized to end-diastolic volume, *LV* left ventricle, *RV* right ventricular. *Inter-ventricular dyssynchrony assessed by difference in time to maximal displacement between RV free wall and LV lateral wall (i.e., ≥44 ms by 95^th^ percentile from healthy controls). †*P* value from Mann-Whitney U-Test.

**Table S5** Correlation coefficient R of 4D flow parameters and right ventricular (RV) remodelling, RV function, six-minute walk test and cardiopulmonary exercise test (CPET) parameters in healthy controls and pulmonary arterial hypertension (PAH).

|  | **RV direct flow, %** | **RV residual volume, %** | **LV direct flow, %** | **LV residual volume, %** | **RV peak systolic KEi_EDV_, µJ/ml** | **RV average systolic KEi_EDV_, µJ/ml** | **RV peak E-wave KEi_EDV_, µJ/ml** | **KE discordance** | **Fractional flow ratio** |
| --- | --- | --- | --- | --- | --- | --- | --- | --- | --- |
| RVEDV/LVEDV ratio | -0.624* | 0.502* | -0.075 | 0.227^§^ | 0.045 | 0.130 | 0.130 | -0.128 | -0.469* |
| RVEF, % | 0.781* | -0.715* | 0.101 | -0.163 | 0.315† | 0.311† | 0.039 | 0.413* | 0.654* |
| Right atrial area, cm^2^ | -0.548* | 0.448* | -0.307† | 0.328† | -0.011 | 0.115 | 0.185 | -0.194 | -0.421* |
| TAPSE, mm | 0.494* | -0.391* | -0.027 | -0.023 | 0.176 | 0.264† | 0.097 | 0.491* | 0.358* |
| RV GLS, % | 0.671* | -0.565* | 0.189 | -0.193 | 0.220^§^ | 0.237^§^ | 0.044 | 0.423* | 0.534* |
| Peak VO_2_, ml/kg/min | 0.424* | -0.450* | 0.109 | -0.078 | -0.020 | 0.029 | -0.057 | 0.223^§^ | 0.363* |
| % predicted peak VO_2_, % | 0.462* | -0.458* | 0.075 | -0.085 | -0.073 | -0.030 | -0.079 | 0.216^§^ | 0.398* |
| VE/VCO_2_ slope | -0.565* | 0.538* | -0.026 | 0.139 | -0.095 | -0.119 | -0.080 | -0.384* | -0.428* |

*LV* left ventricular, *KE discordance* RV/LV systolic KEi_EDV_, *KEi_EDV_* kinetic energy normalized to EDV, *fractional flow ratio* RV direct flow/RV residual volume, *RVEDV* right ventricular end-diastolic volume, *LVEDV* left ventricular end-diastolic volume, *RVEF* right ventricular ejection fraction, *TAPSE* tricuspid annular plane systolic excursion, *GLS* global longitudinal strain, *VO_2_* oxygen uptake, *VE* minute ventilation, *VCO_2_* carbon dioxide output. *Significant level at 0.001; †Significant level at 0.01; ^§^Significant level at 0.05.

**Table S6** Predictors of the prospectively identified events using Cox regression

| **4D flow parameters** | Adj HR (95% CI)* | *P* | Harrell’s C  Statistic |
| --- | --- | --- | --- |
| RV direct flow, % | 0.949 (0.855, 1.053) | 0.326 | 0.651 |
| RV retained inflow, % | 0.928 (0.788, 1.094) | 0.376 | 0.506 |
| RV delayed ejection flow, % | 0.962 (0.803, 1.153) | 0.674 | 0.549 |
| RV residual volume, % | 1.061 (0.976, 1.154) | 0.164 | 0.645 |
| RV peak systolic KEi_EDV_, µJ/ml | 0.990 (0.916, 1.069) | 0.790 | 0.500 |
| RV average systolic KEi_EDV_, µJ/ml | 0.993 (0.853, 1.156) | 0.929 | 0.586 |
| RV peak E-wave KEi_EDV_, µJ/ml | 0.985 (0.909, 1.068) | 0.714 | 0.644 |

*RV* right ventricular, *HR* hazard ratio, *CI* confidence interval, *KEi_EDV_* kinetic energy normalized to end-diastolic volume, *EDV* end-diastolic volume.

*All variables were adjusted for age, sex, and body surface area.

**Table S7** Intra- and interobserver agreement of right ventricular (RV) flow components and kinetic energy parameters.

|  | **Mean ± difference** | ***P*** | **ICC (95% CI)** | ***P*** | **CV** |
| --- | --- | --- | --- | --- | --- |
| **Intra-observer** | | | | | |
| Direct flow, % | 0.26 ± 0.93 | 0.224 | 0.998 (0.996, 0.999) | <0.001 | 2.3 |
| Retained inflow, % | 0.38 ± 1.88 | 0.383 | 0.950 (0.874, 0.980) | <0.001 | 7.8 |
| Delayed ejection flow, % | -0.47 ± 1.38 | 0.146 | 0.977 (0.942, 0.991) | <0.001 | 6.0 |
| Residual volume, % | -0.35 ± 1.04 | 0.149 | 0.998 (0.995, 0.999) | <0.001 | 2.1 |
| Peak systolic KEi_EDV_, µJ/ml | -0.24 ± 0.78 | 0.195 | 0.992 (0.979, 0.997) | <0.001 | 2.6 |
| Average systolic KEi_EDV_, µJ/ml | -0.04 ± 0.62 | 0.786 | 0.991 (0.976, 0.996) | <0.001 | 3.5 |
| Peak E-wave KEi_EDV_, µJ/ml | -0.03 ± 0.45 | 0.811 | 0.999 (0.997, 1.000) | <0.001 | 2.0 |
| **Inter-observer** | | | | | |
| Direct flow, % | 0.08 ± 0.99 | 0.720 | 0.998 (0.996, 0.999) | <0.001 | 2.4 |
| Retained inflow, % | 0.39 ± 2.10 | 0.411 | 0.940 (0.848, 0.976) | <0.001 | 8.7 |
| Delayed ejection flow, % | -0.20 ± 1.92 | 0.648 | 0.958 (0.895, 0.984) | <0.001 | 7.8 |
| Residual volume, % | -0.46 ± 1.09 | 0.077 | 0.998 (0.995, 0.999) | <0.001 | 2.2 |
| Peak systolic KEi_EDV_, µJ/ml | -0.57 ± 1.43 | 0.093 | 0.995 (0.988, 0.998) | <0.001 | 4.9 |
| Average systolic KEi_EDV_, µJ/ml | -0.24 ± 0.74 | 0.171 | 0.991 (0.976, 0.996) | <0.001 | 4.4 |
| Peak E-wave KEi_EDV_, µJ/ml | 0.15 ± 1.03 | 0.528 | 0.998 (0.995, 0.999) | <0.001 | 4.6 |

Mean ± difference between repeated measures and significance were tested with a paired Student T-test and agreement using intraclass correlation coefficient (ICC). *CI* confidence interval, *CV* coefficients of variation, *KEi_EDV_* kinetic energy normalized to end-diastolic volume.
