# Supplementary material for: Right ventricular energetic biomarkers from 4D Flow CMR are associated with exertional capacity in pulmonary arterial hypertension
Source: J Cardiovasc Magn Reson. 2022 Dec 1;24:61. doi: 10.1186/s12968-022-00896-8 (PMC9714144; doi:10.1186/s12968-022-00896-8)
Supplement: Supplementary file 2 — Additional file 2. Methods for 4D flow analysis. [file 12968_2022_896_MOESM2_ESM.docx]

**4D flow analysis**

4D flow images were analyzed using MASS software. Error corrections and 4D flow quality checks were performed as previously reported [1-4]. The same phasic endocardial and epicardial contours generated during ventricular volume analysis were used for 4D flow postprocessing. Positions of 4D flow CMR pathlines in the LV or RV within the endocardial borders at end-systole defined four flow components [5-6]: (1) direct flow: blood that entered and exited the ventricle in the analyzed cardiac cycle; (2) retained inflow: blood that entered the ventricle but did not exit during the analyzed cycle; (3) delayed ejection flow: blood within the ventricle at the start of the analyzed cycle that exited during the analyzed cycle; and (4) residual volume: blood that remained in the ventricle for the duration of at least one full cardiac cycle. The volume of each flow component was indexed to the corresponding ventricular EDV to calculate flow component proportions. For each voxel, KE was computed using the following formula:

$KE=\frac{1}{2}\rho_{\mathrm{blood}}\cdot V_{\mathrm{voxel}}\cdot v_{\mathrm{voxel}}^{2}$,

with ­$\rho_{\mathrm{blood}}$ being the density of blood (1.06 g/cm^3^), $V_{\mathrm{voxel}}$ the voxel volume and $v_{\mathrm{voxel}}$ the velocity magnitude of the corresponding voxel. Total KEs for the LV and RV throughout the cardiac cycle were obtained by summing individual voxel KE values within the ventricular endocardial borders across all time points. All KE parameters were normalized to EDV (KEi_EDV_) and reported in μJ/ml. Phasic KEi_EDV_ parameters (peak systole, average systole and peak E-wave) were extracted from the time-resolved KE curves.

References

1. Crandon S, Westenberg JJM, Swoboda PP, Fent GJ, Foley JRJ, Chew PG, et al. Impact of age and diastolic function on novel, 4d flow CMR biomarkers of left ventricular blood flow kinetic energy. Sci Rep. 2018;8:14436.
2. Barker N, Zafar H, Fidock B, Elhawaz A, Al-Mohammad A, Rothman A, et al. Age-associated changes in 4D flow CMR derived tricuspid valvular flow and right ventricular blood flow kinetic energy. Sci Rep. 2020;10:9908.
3. Zhao X, Tan RS, Garg P, Chai P, Leng S, Bryant J, et al. Impact of age, sex and ethnicity on intra-cardiac flow components and left ventricular kinetic energy derived from 4D flow CMR. Int J Cardiol. 2021;336:105-112.
4. Zhao X, Hu L, Leng S, Tan RS, Chai P, Bryant J, et al. Ventricular flow analysis and its association with exertional capacity in repaired tetralogy of Fallot: 4D flow cardiovascular magnetic resonance study. J Cardiovasc Magn Reson. 2022;24:4.
5. Eriksson J, Carlhäll CJ, Dyverfeldt P, Engvall J, Bolger AF, Ebbers T. Semi-automatic quantification of 4D left ventricular blood flow. J Cardiovasc Magn Reson. 2010;12:9.
6. Eriksson J, Bolger AF, Ebbers T, Carlhäll CJ. Four-dimensional blood flow specific markers of LV dysfunction in dilated cardiomyopathy. Eur Heart J Cardiovasc Imaging. 2013;14:417–424.
