# Supplementary figures and images for: Right ventricular energetic biomarkers from 4D Flow CMR are associated with exertional capacity in pulmonary arterial hypertension
Source: J Cardiovasc Magn Reson. 2022 Dec 1;24:61. doi: 10.1186/s12968-022-00896-8 (PMC9714144; doi:10.1186/s12968-022-00896-8)

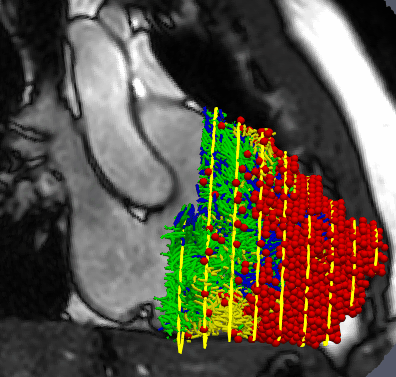

Supplement: Supplementary file 3 — Additional file 3. Movies showing four-chamber views with right ventricle (RV) four flow components using particle tracing in a 48-year-old healthy subject, and a 61-year-old pulmonary arterial hypertension (PAH) patient. Yellow circles denote the RV contours from stacks of short axis views. Color legend: green (RV direct flow), yellow (RV retained inflow), blue (RV delayed ejection flow), red (RV residual volume). [file 12968_2022_896_MOESM3_ESM.zip › Additional file 3/Additional file 3 - Healthy control.gif]

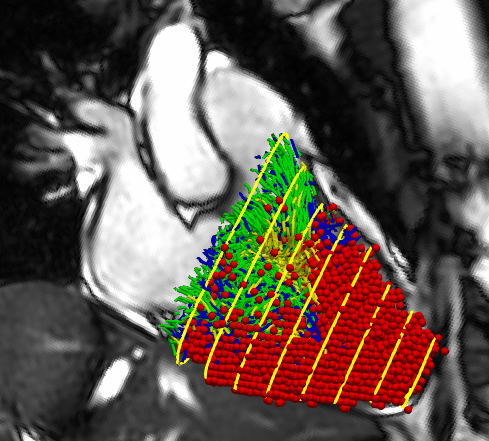

Supplement: Supplementary file 3 — Additional file 3. Movies showing four-chamber views with right ventricle (RV) four flow components using particle tracing in a 48-year-old healthy subject, and a 61-year-old pulmonary arterial hypertension (PAH) patient. Yellow circles denote the RV contours from stacks of short axis views. Color legend: green (RV direct flow), yellow (RV retained inflow), blue (RV delayed ejection flow), red (RV residual volume). [file 12968_2022_896_MOESM3_ESM.zip › Additional file 3/Additional file 3 - PAH.gif]
