# Supplementary material for: Right ventricular energetic biomarkers from 4D Flow CMR are associated with exertional capacity in pulmonary arterial hypertension
Source: J Cardiovasc Magn Reson. 2022 Dec 1;24:61. doi: 10.1186/s12968-022-00896-8 (PMC9714144; doi:10.1186/s12968-022-00896-8)
Supplement: Supplementary file 4 — Additional file 4: Figure S1. Difference in time to maximal displacement between right ventricle (RV) free wall and left ventricle (LV) lateral wall (Time difference = RV-LV) for healthy control (left) and PAH (right). Figure S2. Heat map for the correlation coefficient R of 4D flow parameters and right ventricular (RV) remodelling, RV function, 6MWT and cardiopulmonary exercise test (CPET) parameters in healthy controls and pulmonary arterial hypertension (PAH). Figure S3. Bland-Altman analysis of right ventricular (RV) 4D flow measurements for (A) intraobserver; (B) interobserver for RV direct flow (first row, left), RV retained inflow (first row, right), RV delayed ejection flow (second row, left), RV residual volume (second row, right), RV peak systolic KEiEDV (third row, left), RV systolic KEiEDV (third row, right), and RV peak E-wave KEiEDV (last row). [file 12968_2022_896_MOESM4_ESM.docx]

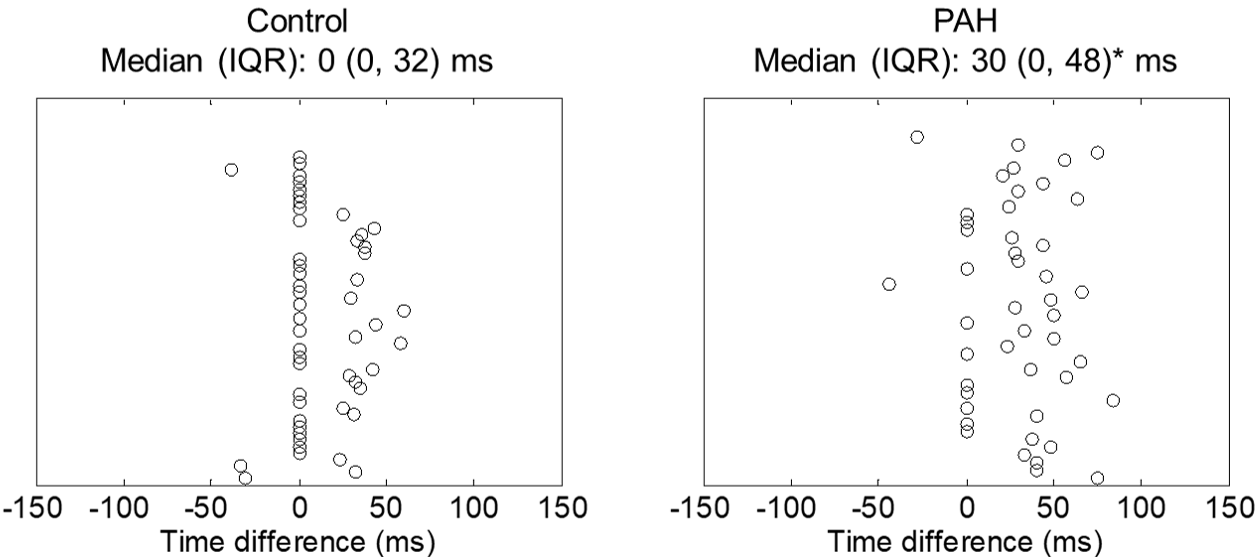


**Fig. S1** Difference in time to maximal displacement between right ventricle (RV) free wall and left ventricle (LV) lateral wall (Time difference=RV$-$LV) for healthy control (left) and PAH (right). *IQR* interquartile range, *PAH* pulmonary arterial hypertension. *Significantly greater than healthy controls.


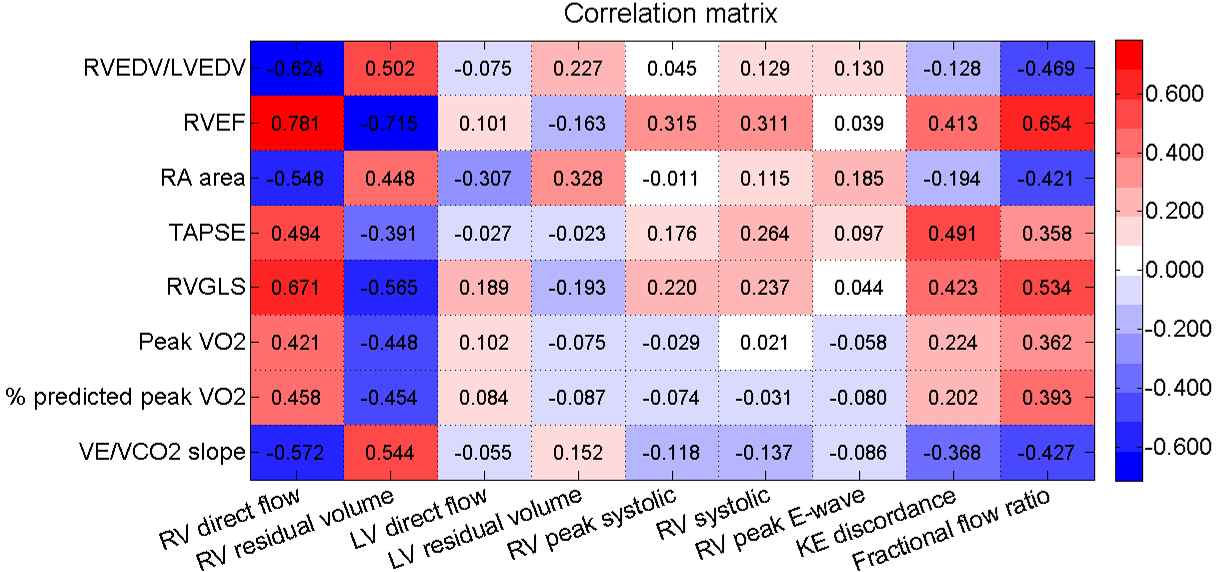


Figure S2. Heat map for the correlation coefficient R of 4D flow parameters and right ventricular (RV) remodelling, RV function, six-minute walk test and cardiopulmonary exercise test (CPET) parameters in healthy controls and pulmonary arterial hypertension (PAH).

**
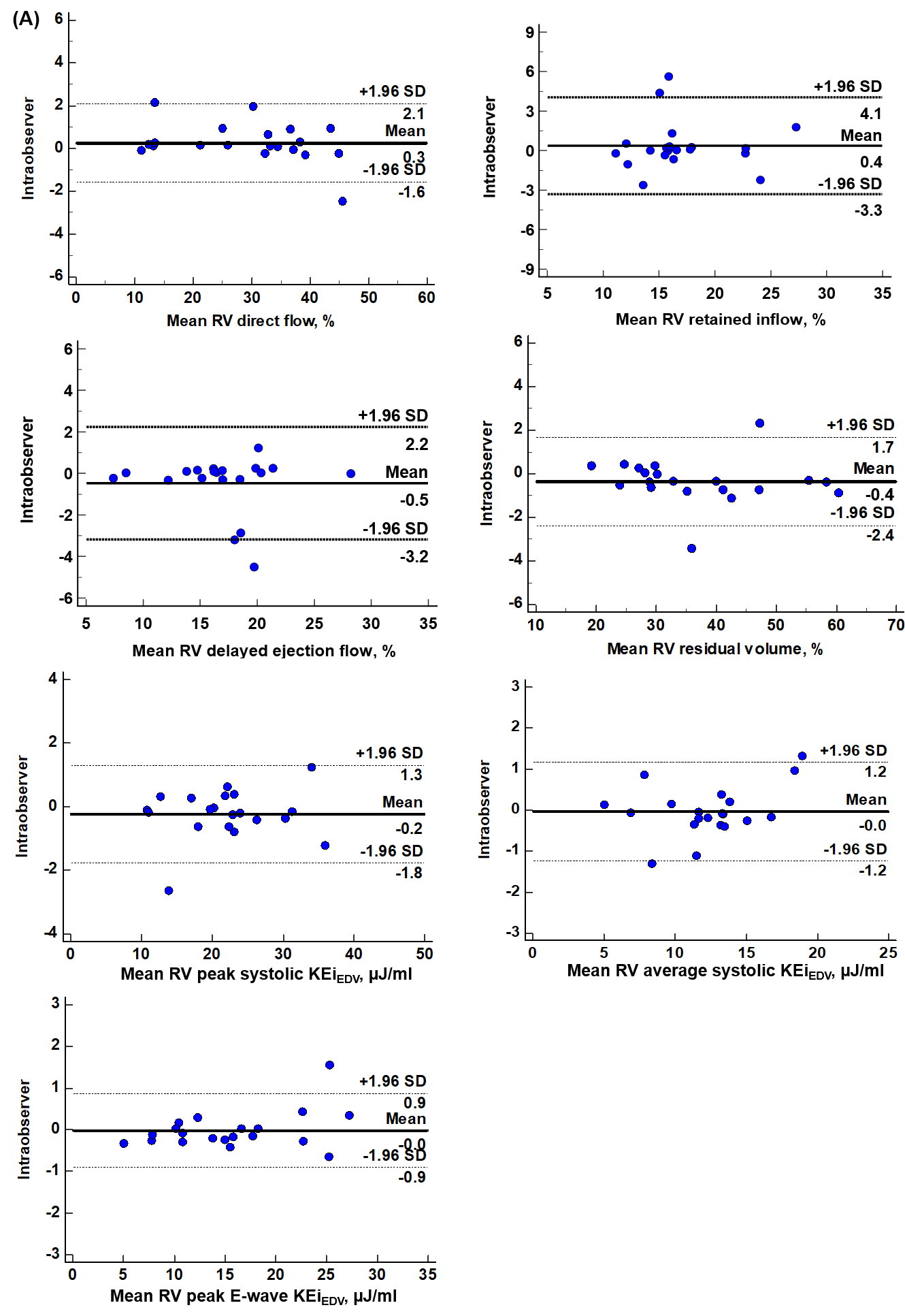

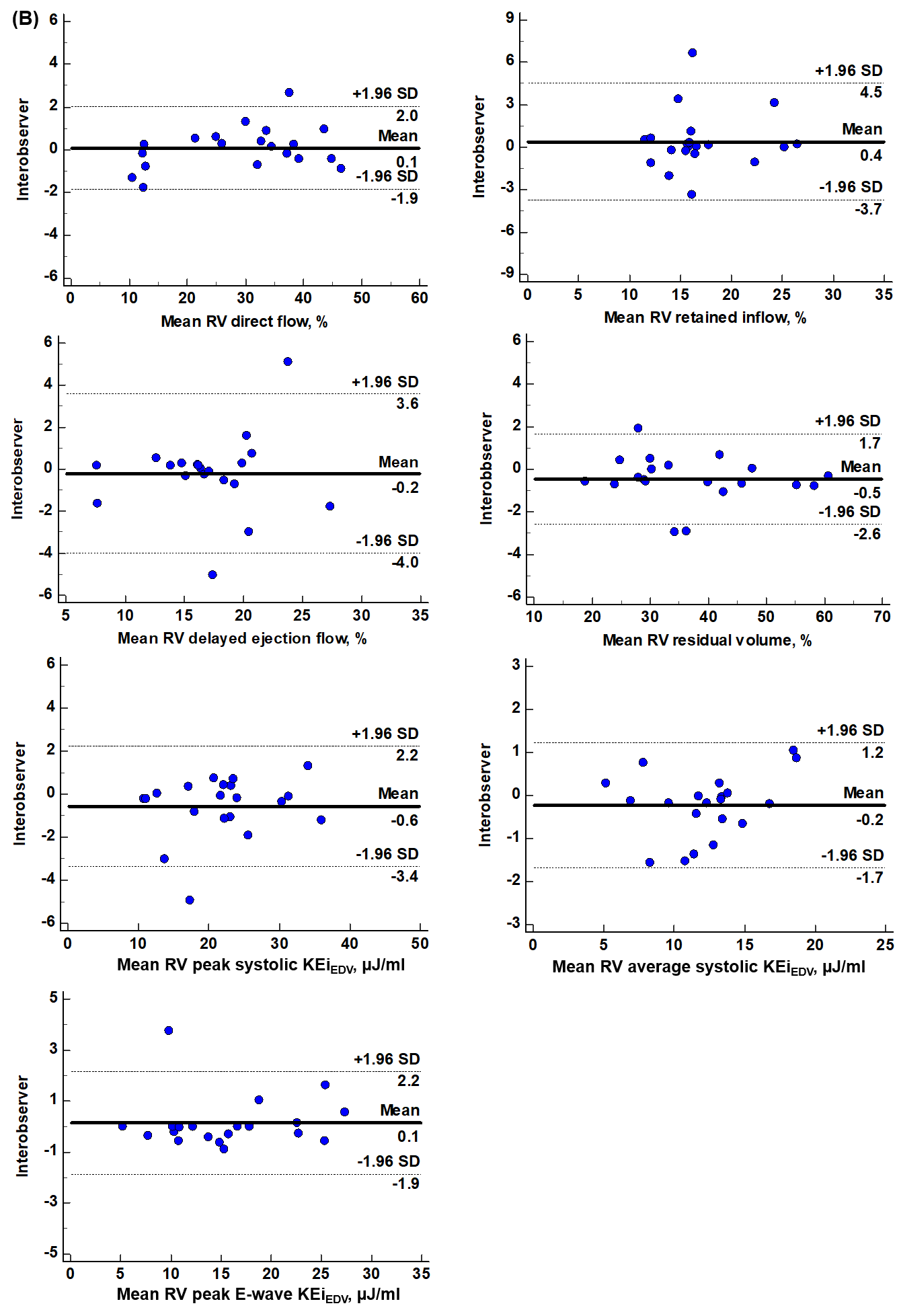
Figure S3.** Bland-Altman analysis of right ventricular (RV) 4D flow measurements for **(A)** intraobserver; **(B)** interobserver for RV direct flow (first row, left), RV retained inflow (first row, right), RV delayed ejection flow (second row, left), RV residual volume (second row, right), RV peak systolic KEi_EDV_ (third row, left), RV systolic KEi_EDV_ (third row, right), and RV peak E-wave KEi_EDV_ (last row). *4D* four-dimensional, *CMR* cardiovascular magnetic resonance, *KEi_EDV_* kinetic energy normalized to end-diastolic volume.
